# In situ X-ray spectroscopies beyond conventional X-ray absorption spectroscopy on deciphering dynamic configuration of electrocatalysts

**DOI:** 10.1038/s41467-023-42370-8

**Published:** 2023-10-18

**Authors:** Jiali Wang, Chia-Shuo Hsu, Tai-Sing Wu, Ting-Shan Chan, Nian-Tzu Suen, Jyh-Fu Lee, Hao Ming Chen

**Affiliations:** 1 Department of Chemistry and Center for Emerging Materials and Advanced Devices, National Taiwan University, Taipei, 10617 Taiwan; 2 National Synchrotron Radiation Research Center, Hsinchu, 30076 Taiwan; 3 College of Chemistry & Chemical Engineering, Yangzhou University, 225002 Yangzhou, China; 4 Graduate Institute of Nanomedicine and Medical Engineering, College of Biomedical Engineering, Taipei Medical University, Taipei, 11031 Taiwan

**Keywords:** Renewable energy, Electrocatalysis

## Abstract

Realizing viable electrocatalytic processes for energy conversion/storage strongly relies on an atomic-level understanding of dynamic configurations on catalyst-electrolyte interface. X-ray absorption spectroscopy (XAS) has become an indispensable tool to in situ investigate dynamic natures of electrocatalysts but still suffers from limited energy resolution, leading to significant electronic transitions poorly resolved. Herein, we highlight advanced X-ray spectroscopies beyond conventional XAS, with emphasis on their unprecedented capabilities of deciphering key configurations of electrocatalysts. The profound complementarities of X-ray spectroscopies from various aspects are established in a probing energy-dependent “in situ spectroscopy map” for comprehensively understanding the solid-liquid interface. This perspective establishes an indispensable in situ research model for future studies and offers exciting research prospects for scientists and spectroscopists.

## Abstract (web summary)

X-ray absorption spectroscopy (XAS) has become an indispensable tool to investigate dynamic natures of electrocatalysts. In this perspective, advanced X-ray spectroscopies are highlighted in a complementary way, providing a promising research model for solid-liquid interface and (electro)catalysis studies.

## Introduction

Increasing environmental concerns coupled with pressing need of sustainable energy economy have brought the studies of electrocatalytic processes that might lead to environmental-friendly chemicals and fuels to the forefront of fundamental and applied research^1^. Notably, making such electrochemical processes technologically viable strongly relies on an atomic-scale understanding of electrocatalyst configurations at the solid–liquid interface^2,3^. Based on empirical and theoretical investigations during past years, a general understanding is that the electrocatalysts undergo dynamic evolutions under working conditions instead of remaining their originally designed states, because they adapt the initial configurations to the varied local environments during reactions^4–6^. Accordingly, in situ/operando identification of dynamic electrocatalyst configurations at the interface is a critical key to understand the catalytic fate for target reactions, which in turn essentially guides effective strategies for designing efficient and promising electrocatalysts^2,7^. However, researchers have been struggling against a long-standing challenge of precisely deciphering atomic-scale configurations of electrocatalysts under realistic conditions due to the substantial conundrum of technologies.

It has to be noticed that, at the electrocatalytic solid–liquid interface, the dynamic catalyst surface with coexisting reactant, intermediate and product species are commonly referred to disordered features rather than ordered crystalline nature. In this regard, only a few characterization manners can practically unravel the dynamic configuration of electrocatalyst at the interface. X-ray absorption spectroscopy (XAS) has become one of the most popular techniques for providing fruitful information regarding the dynamic structures of electrocatalysts without long-range order, wherein X-ray absorption near edge structure (XANES) and extended X-ray absorption fine structure (EXAFS) spectra offer informative features about the electronic structure and coordination environment with elemental specificity, respectively^8^. Although XAS technique is increasingly employed to make significant breakthroughs in electrocatalysis, a reliable data processing and analysis procedure that are vital to accurately interpret spectral features have not been unified in the research community, leading to diverse and even misleading information. To date, quantitative analysis of spectroscopic features in both XANES and EXAFS spectra has still been a challenging task, because these data interpretation is often nontrivial and isn’t well represented in existing literatures. Moreover, another main concern remains that the conventional XAS is still suffering from a remarkable limitation in its poor energy resolution^8,9^, resulting in a fact that many important dynamic features in XANES spectra may not be fully uncovered. Furthermore, for light elements (C, N, and O) that are involved in those reactant, intermediate and product species during various electrocatalytic reactions, the conventional XAS is unable to distinguish them, let alone identify the reactive site–adsorbate interaction configurations^10^. Thus, exploring new opportunities from contemporary X-ray techniques for precisely deciphering the dynamic configurations at the electrochemical interface has become even more pressing.

With the advances of high photon flux at new-generation synchrotron radiation facilities, advanced X-ray spectroscopies with high-energy resolution, such as high-energy-resolution fluorescence-detected XAS (HERFD-XAS), nonresonant X-ray emission spectroscopy (XES) and resonant inelastic X-ray scattering (RIXS), have been rapidly developed in recent years. They provide unprecedented possibilities for probing the electronic excitations and atomic structures of reactive centers, revealing more detailed structure information that is unlikely to be achieved by conventional XAS technique. Particularly, based on hard X-ray irradiation, these techniques can be anticipated to be powerful tools for studying electrochemical processes that are commonly operated at ambient condition, giving significant information on interfacial nature during the rate-limiting steps, which empowers the deciphering of electronic and atomic configurations with presenting significant catalyst-adsorbate interactions at interface. While numerous successful stories have been witnessed in thermal catalysis and biological sciences, as well as enzymatic systems^11^, HERFD-XAS, XES, and RIXS methods remain currently underrepresented in the electrocatalysis field probably owing to the complex behaviors and diverse environments at the electrocatalytic interface. Recently, their great potentials in precisely understanding dynamic aspects in electrocatalysis are increasingly attracting more attentions^9,12^, thus shortening the gap between these promising X-ray spectroscopies and dynamically electrocatalytic behaviors at the solid–liquid interface is highly desired.

Herein, we first convey a few promising/appropriate approaches for the determination of oxidation state from XANES spectra and appeal the attention to the correlation problems of extracted parameters in EXAFS fitting process for complex heteroatomic electrocatalysts, which gives rigorous guidelines in decoding spectroscopic data. Furthermore, we particularly highlight several advanced X-ray spectroscopies from both a spectroscopic (fundamental principles) and instrumental (detection mode) point of view, aiming to open up new possibilities for probing the atomic-level interaction configuration at interface under electrochemical working conditions. An emphasis is placed on the advantages and complementarities of advanced X-ray spectroscopies techniques as compared to the conventional XAS method, along with those key questions about what they can address. Finally, for achieving a comprehensive understanding of potential-driven chemical states and dynamic atomic-configuration evolutions at interface during reactions, a complementary probing energy-dependent “in situ spectroscopy map” is highlighted, which establishes an indispensable research model for electrochemical interfaces and offers a new guideline for future researches on electrocatalysis and beyond.

## Data analysis on XAS spectra

**Fig. 1:**
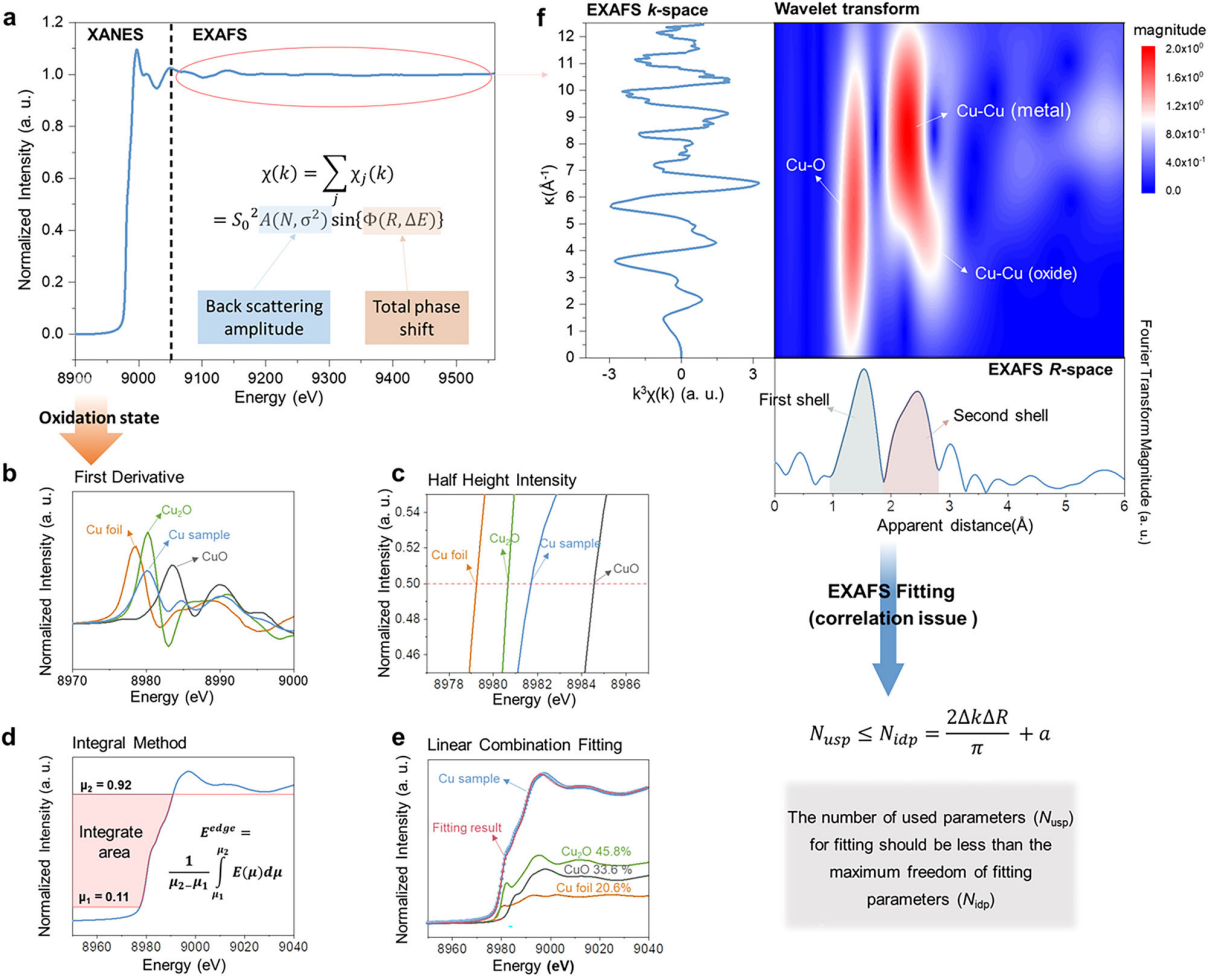
Data analysis on XANES and EXAFS spectra. **a** A typical XAS spectrum consisting of XANES and EXAFS regions, inset is the quantitative parametrization of EXAFS spectra. **b**–**e** Methods of determination of oxidation state from the XANES spectra corresponding to a Cu-based mixture sample: **b** first derivative; **c** half height intensity; **d** integral method; and **e** linear combination fitting. An equation used to define an average edge energy is inserted in (**d**), in which the inverse function *E*(*μ*) of the spectrum is integrated between *μ*_1_ and *μ*_2_. **f** Cu K-edge EXAFS spectrum for a Cu-based mixture sample and its Fourier and wavelet transforms. Nyquist criterion estimating the upper bound of the number of independent variables for EXAFS fitting.

During the last decades, XAS has become a capable and indispensable method to probe the dynamic structures of electrocatalysts without long-range order^13^. An XAS spectrum (the absorption cross section versus incident energy) can be divided into two regions, namely XANES and EXAFS that are defined around and beyond the absorption edge, respectively (Fig. 1a). The XANES spectrum displays remarkable features of electronic transitions from the core levels to unoccupied states, reflecting the electronic structures about frontier orbitals (e.g., oxidation state), which involves the hybridized states caused by adsorbed reactants and intermediates during the electrocatalytic reactions. Moreover, since multiple scattering events greatly contribute to the resulting spectroscopic features in XANES, the XANES spectra can also provide informative insights into the coordination geometry of probed atoms. On the other hand, the EXAFS spectrum that originates from the interference features induced by backscattering photo-electrons of neighboring atoms, is the oscillating part of the absorption signal above the edge and dominated by those single scattering events. EXAFS can accordingly unravel the local coordination environment around absorbing atoms (i.e., coordinated element, coordination number, and interatomic distance).

Notably, achieving reliable evidence in XAS studies strongly relies on the accurate data interpretation of acquired spectra. However, quantitative analysis of XANES and EXAFS spectra is commonly nontrivial and still greatly challenged in the community, a rational approach for decoding spectra is thus critically needed. As for XANES region, generally speaking, the energy of absorption edge (*E*_edge_) can provide a “fingerprint analysis” on oxidation state. In the simplest case, the peak position of the first derivative of XANES spectrum is employed to directly recognize the *E*_edge_ (Fig. 1b). Note that, due to the possible presence of electronic excitations in pre-edge region, such method might cause ambiguous identification of the position of *E*_edge_. To avoid this complexity, the position of half height (0.5) of normalized XANES spectrum is utilized to act as *E*_edge_ (Fig. 1c). The specific oxidation state value, particularly for 3d transition-metal oxides, can be accurately determined from regression lines based on reference samples. Note that the key process in this approach is the normalization of spectrum, thus an inappropriate selection of a position due to existing noises in spectra for normalization process would greatly challenge the precise determination of oxidation state and should be well avoided. A sensible normalization process strongly relies on high-quality data rather than the spectra smoothing, because excessive smoothing usually leads to a degraded energy resolution. An alternative approach is to define an average value of *E*_edge_ in an integral way (equation shown in Fig. 1d), in which all data points in a well-selected region that excludes spectral noise and pre-edge feature are considered (interval between *μ*_1_ and *μ*_2_). By using the integration method, Dittmer et al. have displayed a linear dependence of the *E*_edge_ on oxidation state in inorganic and complex manganese systems^14^. Based on further comparisons, they pointed out that the integration method is more accurate than the half height and the derivatives approaches, because it provides a sensible treatment of complex edge shapes, a high precision with numerous points considered, and a smoothing insensitivity. Nonetheless, the disadvantages are that still two parameters (*μ*_1_ and *μ*_2_) need to be chosen and that the method is not tolerant to normalization inaccuracies. When evaluating the oxidation state through these methods, the most important thing to concern is that, owing to the significant multiple scattering contributions in the XANES region, different coordination structures and symmetry around absorbing atom usually yield marked discrepancies in their shapes of XANES spectra, which results in ambiguities in identifying the position of *E*_edge_. For instance, Guda et al. have recently showed that the position and shape of rising edge of Fe with the same oxidation state are highly sensitive to the geometric details of the absorbing site^15^. As a consequence, the correct identification of oxidation state from XANES spectrum needs to be built on well-defined coordination structures of materials with no significant symmetry changes as compared with reference samples. For a non-uniform system that may include several components, the above-mentioned three methods may fail to precisely determine the oxidation state. In this case, the oxidation state can be obtained by an analysis of linear combination which performs a spectral combination of those spectra from a few known components. As shown in Fig. 1e, by fitting the experimental XANES spectrum with a linear combination of spectra corresponding to each particular component, the relative amount of each specific component can be quantitatively determined. More significantly, since XANES spectra of reference materials with known structure are used in data fitting, the multiple scattering contributions can be well considered. One should note that this approach strongly relies on the correct identification of existing components in samples, and thus requires additional information regarding the existing phases/structures that may be clarified from other characterizations.

As for analysis of EXAFS spectrum, the amplitude of EXAFS oscillation is further obtained by converting the energy (*E*) into photoelectron wavenumber (*k*) for finding the photoenergy that exceeds *E*_edge_ (left panel in Fig. 1f). Through employing a Fourier transformed (FT) implement from *k*-space to *R*-space (lower panel in Fig. 1f), EXAFS spectrum can be preliminarily decoded based on a qualitative comparison between obtained *R*-space spectra and those of reference materials. In addition to FT-EXAFS, another implement, wavelet transformed (WT) method, gives the information about not only what distance exists in the spectrum but also which part it contributes, and thereby leading to a visualization in two-dimensional way (both *R*- and *k*-spaces simultaneously). Such information is very useful for the discrimination of various contributions to EXAFS spectra, especially for a few scattering paths with similar interatomic distance. For instance, a WT-EXAFS spectrum of a Cu-based mixture sample clearly shows that a Cu-Cu scattering can originate from both Cu metal and oxide (middle panel in Fig. 1f), which provides a more straightforward view for neighbor-specific information.

Nonetheless, it is noted that all peaks in both FT- and WT-EXAFS spectra might be a result of the superposition of several single- and/or multiple scatterings, and thus, without conducting a spectral fitting for *k*-space spectrum, an accurate and quantitative analysis cannot be achieved. A quantitative parametrization of EXAFS spectrum is presented in Eq. (1)^16^, one needs to fit the significant parameters that describe the backscattering amplitude (*S*_0_^2^, *N*, σ^2^,) and phase shift functions (*R*, Δ*E*_0_) to match the experimental data.

1 $$\chi \left(k\right)=\mathop{\sum}\limits_{j}\frac{{N}_{j}{S}_{0}^{2}}{k{R}_{j}^{2}}{F}_{j}\left(k\right){e}^{-2{R}_{j}/{\lambda }_{j}\left(k\right)}{e}^{-2{k}^{2}{\sigma }_{j}^{2}}\sin \left[2k{R}_{j}+{\Phi }_{j}\left(k\right)\right]$$

where *N* is the coordination number, *S*_*0*_^2^ represents the amplitude reduction factor, 0.7–1.0, *R* denotes the average interatomic distance, *σ*^2^ is the disorder factor. *S*_0_^2^ is rationally obtained by analyzing EXAFS spectra from references with known phases.

A big challenge in curve fitting refers to the correlation issue among fitting parameters, namely, parameters in pairs of (*N* and σ^2^) and (*R* and Δ*E*_0_) are correlative with each other. Taking a case of Rh-C as an example, once Δ*E*_0_ increases by 10 eV, the *R* value changes by ~0.03 Å^17^. This correlation appears relatively straightforward if the fitting process considers those single scattering paths only. However, once two or more kinds of coordinating atoms are located in a similar distance around target atoms that are indistinguishable by FT- or WT-EXAFS, a multi-shell fitting has to be carried out. In this case, the number of variables increase exponentially with increasing paths in the fitting process and the correlation issue among the fitting parameters becomes quite serious, which poses a great challenge for obtaining individual contributions from various scattering paths. For such correlation issue, one has to pay attention to the maximum freedom of fitting parameters (*N*_*idp*_), which is determined by below Eq. (2)^18,19^.

2 $${N}_{{usp}}\le \,{N}_{{idp}}=\frac{2\Delta k\Delta R}{\pi }+a$$

where *N*_*usp*_ is the number of used parameters, *∆k* denotes the range over which the Fourier transformed window, *∆R* represents the range over which the fit is evaluated, *a* is an integer and suggested to be 2 because of specialty of FT-EXAFS carried out in a finite range.

Accordingly, in a practical fitting process, one cannot employ the number of used parameters (*N*_*usp*_) for fitting more than *N*_*idp*_. To restrict the number of independent parameters, a strategy is to perform a so-called constrained fitting that fixes some fitting parameters to the values of known references. Note that, in a specific coordinated shell, several scattering paths with varying structural disorder (i.e., Debye–Waller factor) would cause structural interference to affect the extraction of interatomic distances, which leads to a fact that the correlation between interatomic distances and Debye–Waller factor (*σ*^2^) is not negligible^20^. Since such effects may cause perturbation in data fitting and give rise to unreasonably physical distances, fitting for many *σ*^2^ parameters may mask the actually structural details and should be mitigated as much as possible. As a result, it can be suggested that, in the constrained refinement, the number of parameters is first reduced by treating a set of scattering atoms as a rigid unit^21^. That is, the used parameter of Debye–Waller factor can be reduced by assuming that chemically similar atoms with a similar distance away from the central atom (i.e., absorbing atom) would be an identical value.

**Fig. 2:**
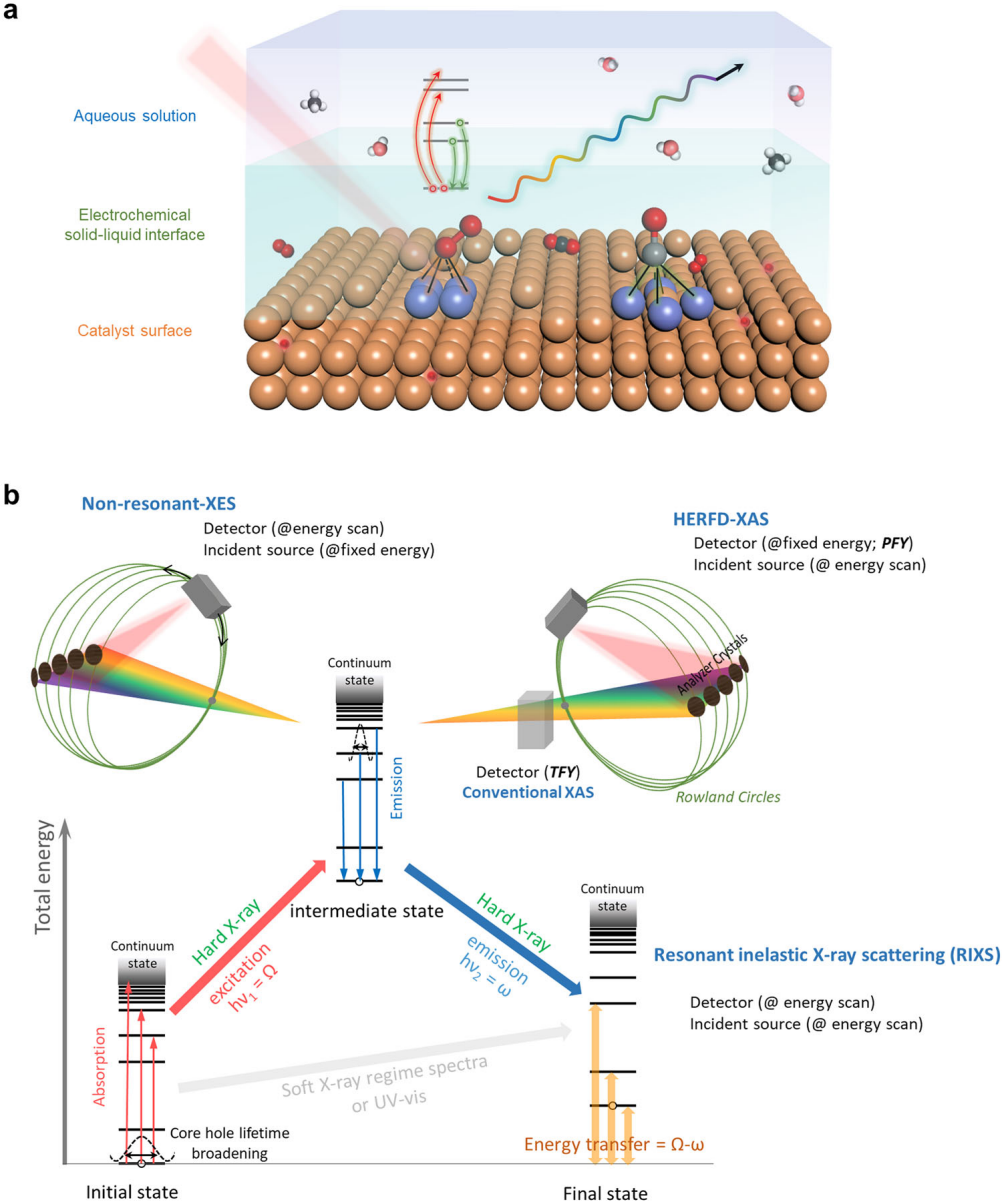
Atomic-scale solid–liquid interface and fundamentals of advanced X-ray spectroscopies. **a** Schematic representation of an electrochemical solid–liquid interface at the molecular level. **b** Total energy schemes for various X-ray photon-in (Ω) and photon-out (ω) spectroscopies, accompanied with their brief fundamentals and experimental schemes for deciphering electrochemical systems.

The EXAFS fitting process that carefully considers the correlation issue would realize a reliable data analysis on extracting informative features from acquired spectra, which then results in an accurate understanding of dynamic configurations on complex heteroatomic electrocatalysts. Importantly, it should be noted that the constrained fitting has to be performed on high-quality spectra, and researchers should dedicate to acquiring qualified data to extract reliable data interpretation. It is not appropriate and should avoid performing the fitting process on poor-quality data. Moreover, for XANES spectra, it is often not easy to obtain an unambiguous interpretation of the observed changes in peak position and intensity, thus appropriate approaches in a specific system must be rigorously selected for achieving accurate data analysis, as discussed earlier in this section. One should note that both XANES and EXAFS spectral analyses need to be performed with caution to avoid overinterpretation of the results. Alternatively, we do strongly suggest conducting multimodal characterizations based on more advanced X-ray techniques to provide more evident features for identifying key configurations (Fig. 2a).

## Emerging opportunities in X-ray spectroscopies for deciphering dynamic configurations

For electrocatalytic processes where the catalytic fate is greatly dominated by a few atomic layers onto the surface of electrocatalysts, judiciously identifying the atomic configuration at the solid–liquid interface after adsorption of key intermediates (as illustrated in Fig. 2a) highly requires sharp features in XAS spectra. Nevertheless, it is noted that the intrinsic broadening of core-hole lifetime leads to a poor energy resolution in conventional XAS spectrum^13^, which results in some important spectral features poorly resolved, especially in its pre-edge and shakedown (known as ligand-to-metal charge transfer) regions that are commonly characteristic of weak features but are strongly correlated with the electronic and geometric configurations of target atoms during reactions. For realizing the enhancement in spectral resolution, a fluorescence-detected mode is preferred^8^. To consider the interaction between X-ray irradiation and core electrons of target elements, specific excitation (absorption)/emission processes may be induced in several ways. Through manipulating both the excitation process and fluorescence signal acquirement, significant spectroscopic features can be ultimately validated to realize various innovative X-ray spectroscopies. A schematic of various photon-in (Ω) and photon-out (ω) processes that are involved in those advanced X-ray spectroscopies is summarized in Fig. 2b.

### High-energy-resolution fluorescence-detected XAS (HERFD-XAS)

Although synchrotron radiation light source has been rapidly developed during past years, it is still unable to offer sufficient energy resolution, because the limit has been the intrinsic lifetime broadening of core hole rather than light source resolution. In principle, for example, after the incident photon with energy Ω = *h*υ_1_ ejects the *K*-shell electron (i.e., 1 *s* electron) to the unoccupied states or into the continuum, the ground state electronic configuration is excited into the intermediate state with the holes in core level (Fig. 2b). The hole re-filled by an outer-shell electron gives rise to the radiative decay of fluorescence with energy ω = *h*υ_2_ or emission of Auger electrons, gaining a final state with a hole in the upper orbital. The Heisenberg uncertainty principle (*Γ* ~ *h*/Δ*t*) provides an energy uncertainty that is inversely proportional to the lifetime (Δ*t*)^1,22^. Accordingly, the intermediate state in this process involves an inner-shell hole with a finite lifetime, leading to a Lorentzian broadening *Γ* of the emission line. The effective lifetime width is determined by the Eq. (3)^23^:

3 $$\varGamma ({{{{{\rm{effective}}}}}}\; {{{{{\rm{lifetime}}}}}}\; {{{{{\rm{width}}}}}})={\varGamma }_{{{{{\mathrm{int}}}}}.}{+\varGamma }_{{fin}.}$$

where ${\varGamma }_{{{{{\mathrm{int}}}}}.}$ and ${\varGamma }_{{fin}.}$ are the core-hole lifetime width of intermediate and final state, respectively. For instance, for a *K*α_1_ emission line, the effective lifetime width of *Γ(K*α_1_*)* should be sum of *Γ*(1 *s*) and *Γ*(2*p*_3/2_). Energy width as a result of the core-hole lifetime broadening normally increases almost exponentially as a function of atomic number (*Z*)^23–25^, and generally, the *L*_*2*_- and *L*_*3*_-shell lifetime width are much sharper (longer lifetime) than that of *K*-shell. As demonstrated, energy width of the *K*-shell varies from 1 eV (Vanadium) up to 40 eV (Tungsten), while those of the *L*-shell range from 3.7 eV (Neodymium) to 7.4 eV (Uranium)^24^.

To suppress the effects of core-hole lifetime broadening, a high-resolution spectrometer (an energy or wavelength dispersive spectrometer that can discriminate among decay channels) should be employed for selecting an interested radiative fluorescence channel that has a smaller lifetime broadening for further improving the energy resolution of resulting spectra. Typically, by equipping an additional crystal analyzer on Rowland geometries^26^, it is able to achieve a typical energy resolution below 1 eV^27^. After the selection process by using a crystal analyzer, the apparent core-hole lifetime broadening can be given by the following Eq. (4)^28^:

4 $$\varGamma \left({{{{{\rm{apparent}}}}}}\; {{{{{\rm{lifetime}}}}}}\; {{{{{\rm{width}}}}}}\right)=\frac{1}{\sqrt{\frac{1}{{\varGamma }_{{{{{\mathrm{int}}}}}.}^{2}}+\frac{1}{{\varGamma }_{{fin}.}^{2}}}}$$

In this sense, with an emission spectrometer in a fixed energy (i.e., selected radiative decay), the fluorescence yield can be integrated over a narrow range centered on a given fluorescence line, which allows to acquire the high-resolution XAS spectra in a partial fluorescence yield (PFY) mode instead of the total number counting (total fluorescence yield; TFY), namely the so-called high-energy-resolution fluorescence-detected XAS (HERFD-XAS). For instance, by using an emission spectrometer to select specific radiative fluorescence channel from *L*-shell (${K}_{\alpha }$ line) or *M*-shell (${K}_{\beta }$ line), the lifetime width *Γ* (1 *s*) can be mitigated to reduce the spectral lifetime width of XAS spectrum. As a result, an absorption spectral sharpening is realized without background contributions basically, which is peculiarly paramount for electrocatalytic investigations with the presence of strongly scattering from liquid environment. Most notably, by using hard X-rays, HERFD-XAS is one of the most powerful techniques for studying interfacial electrocatalysis under ambient conditions.

Owing to a two- to five-fold improvement in energy resolution, the HERFD-XAS enables access to informative features in its pre-edge and shakedown regions that are resolved scarcely through the conventional XAS (with the TFY mode). HERFD-XAS gives a more precise discrimination of the dynamic oxidation state^29^, geometrical symmetry^30^ and chemical bonding (metal–ligand charge transfer) of reactive sites^31^, which allows to reveal the interfacial electrocatalyst-adsorbate interactions during reactions. However, one should note that the HERFD-XAS data collection requires an intensive irradiation source because the fluorescence reaching the detector is greatly suppressed by employing a crystal analyzer, leading to long acquisition time to be used and thus high dose exposure of the sample, which might induce beam damage, especially with the presence of an electrolyte. Therefore, the acquisition of high-quality HERFD-XAS spectra stringently requires an optimized sample condition without undesired irradiation damages.

**Fig. 3:**
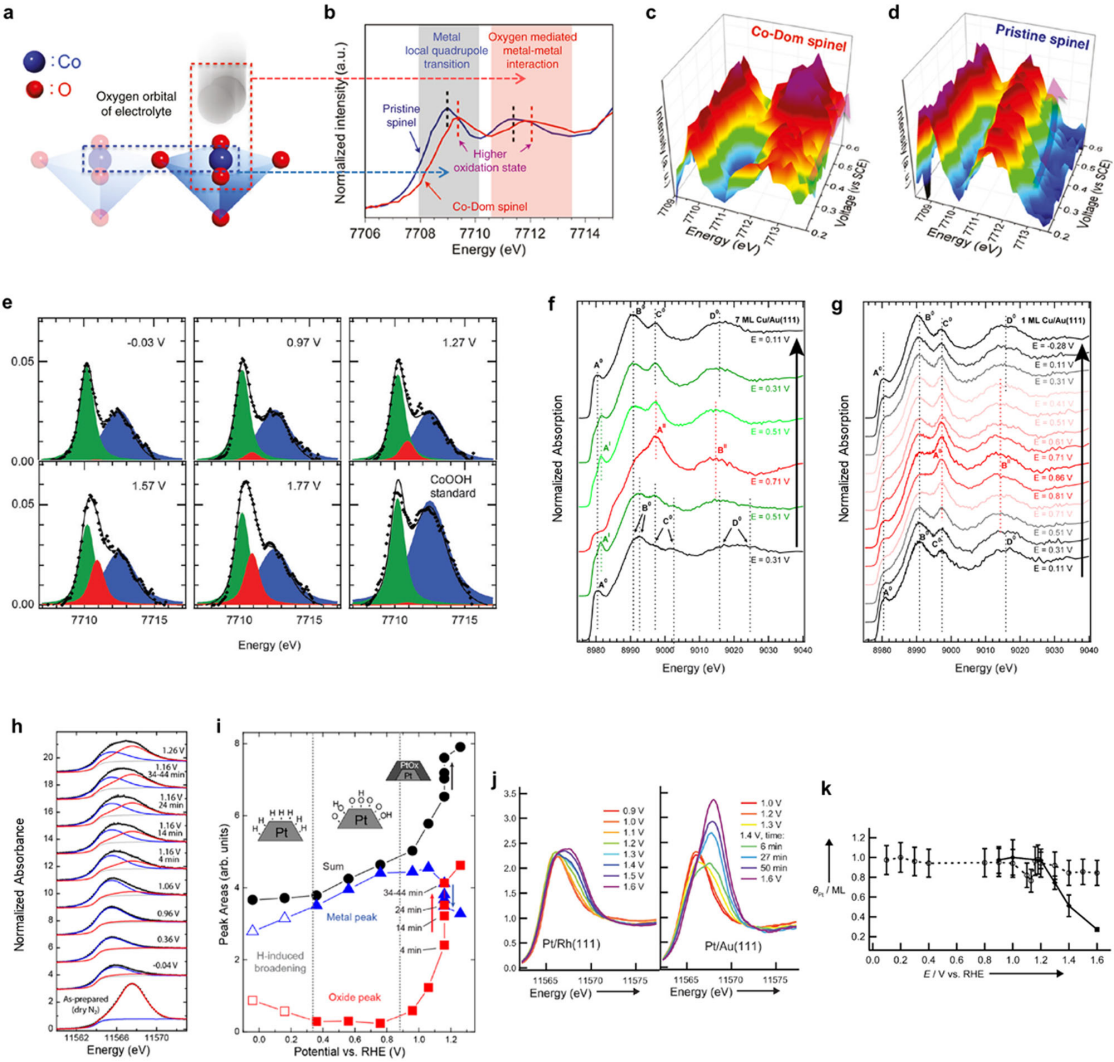
Operando HERFD-XAS analysis on various electrochemical processes. **a** Schematic illustration of interactions between cobalt 3*d* orbital of catalytic surface with oxygen 2*p* orbital of electrolyte. **b** Co *K*-edge HERFD-XAS spectra for pristine spinel and Co-Dom spinel. **c**, **d** Operando Co *K*-edge HERFD-XAS spectra for **c** Co-Dom spinel and **d** pristine spinel during OER. **e** Operando Co *K*-edge HERFD-XAS pre-edge features of Co oxide/Au(111) along with CoOOH reference. **f**, **g** Operando Cu *K*-edge HERFD-XAS spectra of Cu/Au(111) with different Cu coverages of **f** 7 ML and **g** 1 ML as a function of potentials. ML monolayer. Characteristic spectral features are labeled A^0^/B^0^/C^0^/D^0^ for Cu^0^, A^I^ for Cu_2_O, and A^II^/B^II^ for CuO. **h** Least-squares fits to operando Pt *L*_3_ HERFD-XANES spectra of Pt nanoparticles on glassy carbon. **i** Corresponding areas of the fitted components for the full set of operando Pt *L*_3_ HERFD-XANES spectra, as well as their sum. **j** Operando Pt *L*_3_ HERFD-XAS spectra for Pt/Rh(111) (left panel) and Pt/Au(111) (right panel) in 0.01 M HClO_4_. **k** Pt coverage *θ*_Pt_ determined from relative fluorescence count rates at 11,600 eV incident energy as a function of increasing potentials. Pt/Rh(111) is labeled dashed lines with open circles and Pt/Au(111) is labeled solid lines with diamonds. Figures adapted with permission from: **a**–**d** ref. ^32^, American Chemical Society (2018); **e** ref. ^33^, RSC (2013); **f**, **g** ref. ^34^, American Chemical Society (2014); **h**, **i** ref. ^36^, American Chemical Society (2012); **j**, **k** ref. ^37^, Wiley (2011).

Typically, a benefit of in situ HERFD-XAS is the ability to accurately identify the dynamic electronic interaction and geometric environment around active metal sites during various reactions. For instance, the dynamic evolution of *d* orbitals in central metal sites in binary Co–Fe oxides during OER can be directly probed by operando HERFD-XAS with a small incident angle^32^. As illustrated in Fig. 3a, b, Co K-edge HERFD-XAS spectra display two distinct regions in the pre-edge domain: (i) low-energy region, referring to a metal local quadrupole transition; (ii) high-energy region, showing an oxygen-mediated metal-metal interaction. A high-energy shift in HERFD-XAS spectrum for Co-Dom spinel (Fe-doped Co oxide with Co-dominated lattice frame) suggests that the Fe ions significantly influence the oxidation behavior and/or coordination environment of Co ions. In situ Co *K*-edge HERFD-XAS spectra exhibit that, with increasing the applied potentials, the peaks regarding the oxygen-mediated metal-metal interaction in the Co-Dom spinel sample clearly reveal a steady energy position and a substantial increase in intensity as compared with those in the pristine spinel, which verifies an intense orbital interaction between oxygen (2*p*) and cobalt (3*d*) in Co-Dom spinel (Fig. 3c, d). Regarding Fe ions, the HERFD-XAS spectra show the imperceptible differences with increasing the applied potential, implying that iron ions are not involved in the catalytic cycles. Accordingly, in situ HERFD-XAS analysis demonstrates that Co ions serve as the active sites for OER, while Fe ions can effectively stabilize the Co ions to afford higher oxidation states during OER and promote the catalytic interaction between Co ions and electrolyte, leading to a stable intermediate of reactant and then superior intrinsic OER activity.

The HERFD-XAS technique also allows to unambiguously distinguish the changes in surface state and redox phase on electrocatalysts during reactions. For example, operando HERFD-XAS method has been utilized to clearly track the subtle potential-induced changes in the pre-edge features of Co *K*-edge spectra on Co oxide/Au(111) during OER (Fig. 3e)^33^, which are inaccessible to be observed in conventional XAS manner. By performing least-squares fits, it conclusively demonstrates that a new feature at 7710.9 eV in addition to two CoOOH features can be ascribed to the presence of Co^4+^ ions in the H_1-x_CoO_2_ phase at high potentials, which is consistent with the shift of the main absorption edge toward high energy during OER. The appearance of Co^4+^ is validated to be rather detrimental for the OER performance. In another typical work^34^, operando Cu *K*-edge HERFD-XAS spectra with well-sharpened features fully verify that, at high potentials, a monolayer Cu on Au(111) undergoes a direct phase transition from metallic Cu^0^ to CuO rather than forming the Cu_2_O intermediate observed in multilayer Cu/Au(111) (Fig. 3f, g). At low potentials, spectra analysis shows that the Cu monolayer in its metallic state exhibits a lattice expansion because of presence of Au substrate (forming Au-Cu alloy and then Au-terminated surface during its long-term operation), which is expected to significantly affect the interactions between catalytic surface and intermediates for CO_2_ electroreduction reaction (CO_2_RR). Recently, a HERFD-XANES analysis on nanostructured Cu catalysts toward CO_2_RR has provided significant insights on structural factors and Cu^+^ species in facilitating the C_2+_ product formation^35^. However, it also reported that HERFD-XANES spectra suffer from a significant self-absorption effect, which is an important factor to consider in X-ray absorption measurements in fluorescence mode. For thick and concentrated samples, the energy-dependent attenuation of the incident and emitted X-ray photons leads to distortions of the spectral features. Thus, for achieving correct HERFD-XAS spectrum, one should be very careful to choose sample concentration in a way such that the self-adsorption can be avoided. Furthermore, operando HERFD-XAS at Pt *L*_3_ edge is employed to distinguish the chemisorbed hydrogen, chemisorbed oxygen/hydroxyl, and various platinum oxides on the cathode of proton exchange membrane fuel cell (PEMFC) (Fig. 3h, i)^36^. Least-squares fitting results of potential-dependent HERFD-XAS spectra indicate that the observed increases in white-line intensity at high potentials in fact originate from Pt oxides formation rather than previously proposed oxygen-containing species chemisorption. Moreover, as compared to the Pt/Rh(111), a sharp increase of white-line intensity for Pt *L*_3_ HERFD-XAS spectra can be seen for Pt/Au(111) at 1.4 V versus RHE (Fig. 3j)^37^, coinciding with the Pt dissolution at the same potential (Fig. 3k), which implies that the rapid oxide growth in Pt/Au(111) is facilitated by an anodic Pt dissolution to Pt^2+^ with subsequent further oxidation of Pt^2+^ to Pt^4+^. By contrast, the anodic polarization would lead to a passivation on Pt/Rh(111), showing the weakened degradation process during oxygen reduction reaction (ORR).

It should be noted that the HERFD-XAS is mostly utilized to sharpen XANES spectrum instead of EXAFS one in most electrochemical studies, because HERFD-EXAFS with a large energy range suffers from significantly longer data collection times, which is inappropriate to track those transient structures during electrochemical reactions. However, for in-depth studies on the electrocatalytic solid–liquid interface, high-quality EXAFS spectra in an acceptable duration are still highly desired to ideally discriminate the spectral features. For instance, if a catalyst contains several elements having absorption edges near the absorption edge of the target element of interest, the EXAFS spectrum of the target element can be easily distorted by the presence of the coexisting elements, thus an appropriate detection system is indispensable. To this end, an energy-filtered detection scheme is imperatively proposed (vide infra).

### Energy-filtered EXAFS

**Fig. 4:**
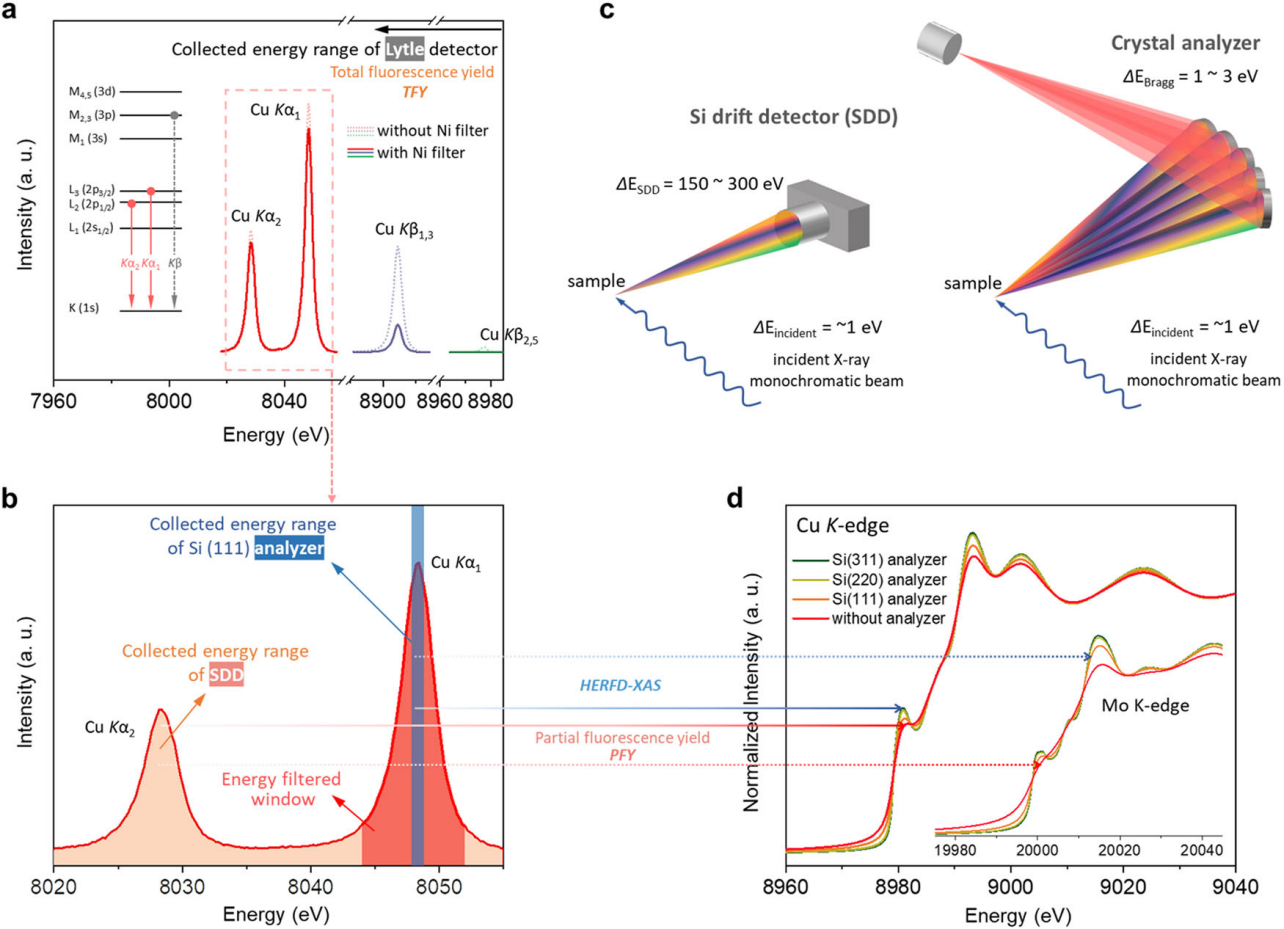
Schematic process of fluorescence detection systems. **a**, **b** Spectra correspond to the analysis of a Cu foil with **a** a Lytle detector (TFY) and with **b** a silicon-drift detector (PFY, orange highlight; Energy-filtered window, red highlight) and a crystal analyzer using Si (111) (HERFD-XAS, blue highlight). In (**a**), note that the nickel filter greatly suppresses Cu *K*β radiation. Inset in (**a**) shows the electronic energy levels for a copper atom. **c** Schematic of fluorescence detection by Si drift detector and Bragg crystal analyzer. **d** Cu *K*-edge XAS spectra of a Cu foil acquired using a Si drift detector and an additional single crystal analyzer (Si (111), Si (220) and Si (311)), inset shows Mo *K*-edge XAS spectra of a Mo foil with a silicon-drift detector and various crystal analyzers.

Figure 4a, b shows the schematic process of fluorescence detection systems. A variety of detectors can be used to collect fluorescence data. A Lytle detector is utilized to collect all emitted fluorescence photons whose energy is below the absorption edge of the selected filter (Fig. 4a), leading to the total fluorescence yield (TFY) XAS spectrum. For instance, with the assistance of a nickel filter that is effective in suppressing the Cu *K*β emission (blue and green lines in Fig. 4a), the Lytle detector realizes the spectra acquisition of a relatively clean beam of *K*α radiation (red line in Fig. 4a). The Lytle detector collects photons regardless of their energy with a bandpass filter only. As an alternative, one can use an energy-resolved detector to minimize those undesired contributions mainly from elastic and those fluorescence photons emitted from other channels. Thanks to rapid development of silicon-drift detector (SDD), in various state-of-the-art XAS beamlines, the SDD with a typical energy resolution of 150–300 eV has been utilized to substitute for Lytle detector (Fig. 4c)^38^. SDD enables the emitted photons to be collected and later electronically separated by energy, which allows one to integrate the fluorescence photons within an energy region of interest (dashed box in Fig. 4a and highlighted in orange at Fig. 4b) to detect a partial fluorescence yield (PFY), giving rise to the resulting spectrum with minimized undesired signals.

It has to be pointed out that the collecting energy range of SDD is significantly larger than that of spectrometer in HERFD-XAS, which can offer an advantage of shortening the data acquisition duration of XAS spectrum. Thus, utilizing SDD is paramount and useful to realize a high-quality EXAFS spectrum for electrochemical systems, which can significantly improve the elemental differential by tuning its energy-filtered window of SDD for matching the emission line of particular element (e.g., using a multi-element SDD aligned at selected maximum region of metals *K*α_1_ emission line, as highlighted in red at Fig. 4b), and discriminate adjacent elements in Periodic Table. Generally, utilization of such energy-filtered fluorescence detector can effectively suppress the interference from undesired elements in leading to an energy-filtered EXAFS spectrum, which is peculiarly significant for atomically dispersed electrocatalysts or discrete sub-nano clusters that put stringent requirements for identifying the metal-metal scattering pairs and interatomic distance during electrocatalysis. Thus, special attention should be paid to the energy-filtered fluorescence measurements for performing operando EXAFS in such new type of electrocatalysts that are commonly hard to be precisely characterized by conventional XAS technique.

Nevertheless, even if a SDD can be utilized to sharpen the XAS spectrum, Compton scattering is still dominating the background signals and the energy-filtered window is still insufficient for providing high-resolution energy-dependent XAS spectra. A similar concept as illustrated in HERFD-XAS can be conducted to sharpen the resulting spectrum by employing additional crystal analyzers positioned on the Rowland circle (Fig. 4c). The whole-crystal surface meets the Bragg diffraction conditions for a certain energy, and the diffraction photons can be focused on the detector. The usage of crystal analyzers provides an implement to integrate a narrow region of target fluorescence line as compared with SDD (highlighted in blue at Fig. 4b), and thus PFY-XAS spectrum with a high-energy resolution, namely HERFD-XAS, can be obtained as well. For example, the sharpening effect on the spectrum by HERFD-XAS can be evidently demonstrated by analyzing XANES spectra of Cu and Mo foils (Fig. 4d). Under several crystal analyzers with different energy resolution, the spectral features in HERFD-XANES spectra can be clearly clarified as compared with those in conventional PFY-mode (using SDD without a crystal analyzer), which greatly improves the accuracy of quantitative XANES analysis. Also, it is further validated that such sharpening effect is more pronounced in 4*d* transition metals than those of 3*d* metals, as showed in the inset of Fig. 4d. Moreover, with the peculiar implementation of crystal analyzer, it is possible to realize the valence-, spin- and ligand-selective XAS by tuning the energy-filtered window to probe specific fluorescence emission line, because the energy of fluorescence lines (especially for *K*_β_ lines) is sensitive to the valence/spin state (*K*_β1,3_) and ligands (*K*_β2,5_) of interesting elements^22,39,40^. Furthermore, through employing the crystal analyzer, other high-resolution X-ray spectroscopic techniques such as XES and RIXS are correspondingly achieved (detailed discussions in following sections).

### Nonresonant X-ray emission spectroscopy (XES)

Because of the exceptional element-specific information from emitted fluorescence, X-ray emission spectroscopy (XES) can be directly realized by using a high-energy-resolution spectrometer as introduced in HERFD-XAS method. Generally, the energy of incident radiation largely exceeds those absorption edges of target elements, the fluorescence emission is independent of incident energy, which is specifically referred to as nonresonant XES, in a contrast with the resonant inelastic X-ray scattering (RIXS). In the resonant case of RIXS, the energies of both incident radiation and radiative fluorescence are simultaneously scanned as the incident energy is tuned across the target absorption edge, by which the electronic states are resonantly excited and emitted to reveal the energy transfer (i.e., energy loss) in between (Fig. 2b). In contrast, the nonresonant XES spectrum is achieved by scanning the energy-dependent fluorescence profile under an incident X-ray with fixed energy. Compared with XANES which describes the electron population of unoccupied states, nonresonant XES provides a highly complementary information regarding the occupied electronic states^41^. *K* line emission that results from re-filling of 1 *s* holes is the most common case because of its relatively high fluorescence yield for studying dilute electrocatalyst samples. Among several refined emission lines, the core-to-core *K*_β_ lines (3*p* to 1 *s* transition), featured with *K*_β1,3_ (main peak) and *K*_β_ʹ (broad shoulder at lower energy), can provide an indispensable probe for unpaired electron numbers (spin state) and bonding covalency of 3*d* transition metals^27^. Moreover, the valence-to-core *K*_β_ spectrum, called *K*_β2,5_ and *K*_β_ʺ, is also of interest because it is rather sensitive to the occupied valence orbitals that are participating in chemical bonding, which can evidently validate the ligand identity and its protonation/hybridization state^11,42^. Particularly, in contrast with EXAFS, valence-to-core *K*_β_ spectra exhibit an overwhelming ability in distinguishing among C-, N-, O-, and F-ligands in the first coordination sphere of the probed atoms that are commonly involved in the formation of covalent bonding during electrocatalysis^22^. Most significantly, although conventional *L*_*2,3*_-edge XAS (transition from *p* to *d* state) of 3*d* transition metals can also directly probe the characters of valence electrons, the nonresonant XES measurement using hard X-rays allows us to elucidate the 3*d* orbital characteristics through its *K*-edge spectrum in ambient conditions, in contrast with that soft X-ray XAS that requires high vacuum chamber^43^. As a consequence, nonresonant XES can provide a precise evaluation of the spin state^44^, electronic interactions^45^, metal–ligand covalency^46^, and meanwhile, give insightful information on the ligand identity and environment^47^, which is even more practical for studying solid–liquid interface of electrocatalytic system that is commonly operated at ambient condition^42^.

**Fig. 5:**
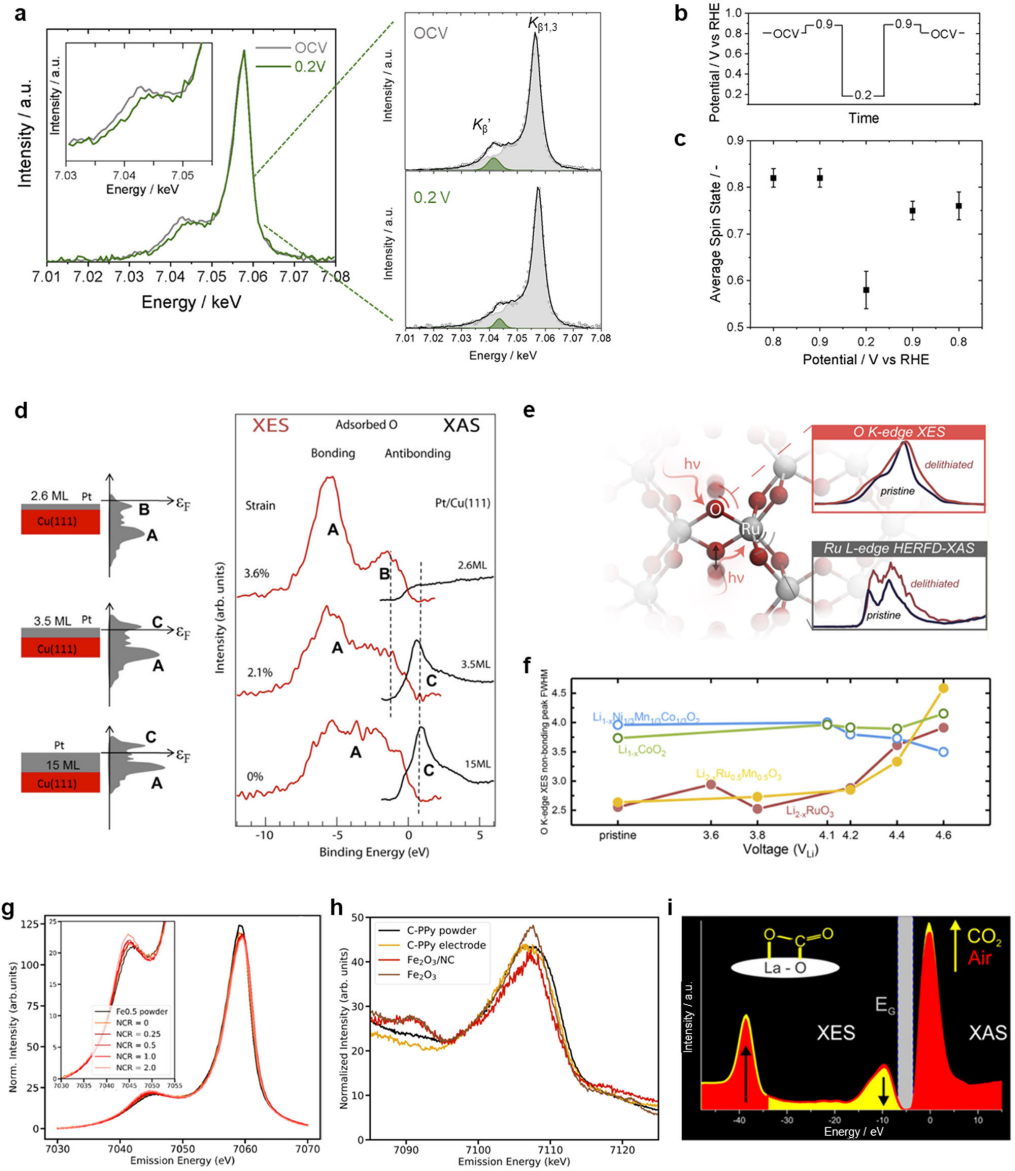
XES analysis on revealing spin/electronic state and binding nature. **a** Comparison of in situ *K*_β_ mainline XES spectra recorded on DW21 catalyst in N_2_-saturated 0.5 M H_2_SO_4_ at open circuit (OCV) and 0.2 V versus RHE, along with corresponding fit results. Inset shows a magnification of the *K*_β_’ region. **b** Scheme of the experimental protocol applied in the in situ XES measurements, whereby each potential hold lasted 10 min. **c** Average spin states at various potentials estimated by interpolation of the spectral *K*_β_’ relative area in the regression line. **d** Pt overlayers of varying thicknesses on a Cu(111). Left: schematic illustration; middle: the qualitatively predicted d band structure; right: oxygen *K*-edge XAS and XES (0.2 ML of oxygen chemisorbed). **e** Schematic illustration of Li_2−*x*_RuO_3_, together with oxygen *K*-edge XES and Ru *L*_3_-edge HERFD-XAS spectra of pristine and delithiated Li_2−*x*_RuO_3_. **f** Nonbonding peak full width at half-maximum (FWHM) of different positive electrode materials as a function of charging potential. **g** Fe *K*_β_ XES spectra recorded on single-atom Fe0.5 catalyst powder and its electrode with the increase of Nafion content. The inset shows magnified *K*_β_’ region. **h** Valence-to-core XES spectra for C-PPy catalyst powder and its electrode as well as iron oxide references. **i** In situ XAS and XES spectra before (red) and after (yellow) exposure to 10,000 ppm of CO_2_ in 50% rh at 250 °C. Inset is schematic of possible carbonate species forming at the surface of La_2_O_2_CO_3_. Figures adapted with permission from: **a**–**c** ref. ^48^, Wiley (2021); **d** ref. ^51^, Elsevier (2016); **e**, **f** ref. ^52^, American Chemical Society (2019); **g**, **h** ref. ^53^, American Chemical Society (2023); **i** ref. ^54^, American National Academy of Sciences (2015).

Despite their applications mainly observed in biological catalysis^11^, these emission lines have recently found utility in electrocatalysis. One of few examples of the nonresonant XES approach in electrocatalysis is tracking of dynamic spin state changes during electrochemical reactions. By performing in situ XES measurements under oxygen electroreduction condition, the potential-induced spin state variations in Fe/N/C catalysts have been well-revealed^48^. Operando XES spectrum recorded at 0.2 V versus RHE displays a smaller *K*_β_ʹ feature with relative to that of measured at OCV (Fig. 5a), indicative of a decrease in average spin state of iron atoms during ORR. A further quantitative analysis shows that a potential decrease from OCV/0.9 to 0.2 V versus RHE leads to an average spin state drop from 0.8 to 0.55, and the spin state immediately reverses once high potentials are applied (Fig. 5b, c). Such spin state transition observed by in situ XES method might correlate with the changes in the atomic configuration of Fe-N sites proposed in previous in situ XAS studies for Fe/N/C systems^49^, which essentially governs the ORR efficiency. Taking advantage of the spin and oxidation state sensitive *K*_β1,3_ peak obtained by using XES, the cation site occupation in complex spinel systems, an important determinant of electrocatalytic properties, can be determined. Taking a recent work as an example^50^, the spinel phase Co_x_Mn_3-x_O_4_ nanoparticles contaminated with CoO are investigated because Mn and Co can occupy all cation sites and the impurity simulates typical products of oxide formation. By a linear combination analysis on the normalized Co- and Mn-*K*_β1,3_ spectra with those of bulk references, the concentrations of all existing cation species at their respective sites (${{Co}}_{{T}_{d}}^{2+}$, ${{Co}}_{{O}_{h}}^{2+}$, ${{Co}}_{{O}_{h}}^{3+}$, ${{Mn}}_{{T}_{d}}^{2+}$, ${{Mn}}_{{O}_{h}}^{2+}$ and ${{Mn}}_{{O}_{h}}^{3+}$) are determined. However, such discrimination of cation oxidation state is not achievable through the widely used *K*-edge XAS method. In fact, the high errors from the many assumptions employed in conventional XAS limit the accuracy of extracted occupation information. It reveals that XES is a superior and a far more accurate method than XAS in extracting cation site occupation in spinel crystal structures.

Moreover, nonresonant XES approach shows an advantage of directly monitoring changes in the electronic interactions during reactions. In a typical work, the *K*-edge XES and XAS spectra of atomic oxygen adsorbed on Pt overlayers on Cu(111) substrate directly reveal the position and occupation of the oxygen 2*p* and Pt 5*d* antibonding states projected onto the oxygen atom during ORR (Fig. 5d)^41,51^. For oxygen on the thickest Pt overlayer, a broad occupied bonding state in the XES spectrum (feature A) was found with an intense resonance related to the antibonding state (feature C) in the XAS spectrum. With decreasing the overlayer thickness, a lattice strained thin Pt overlayer exhibits additional XES feature B and vanishing XAS feature, demonstrating a downshift of the oxygen projected bands. For Pt overlayer with maximum strain, it can be seen that the antibonding state is fully occupied with a peak around 1.5 eV below the Fermi level. These results strongly validate the fundamental origin of the lower chemisorption of adsorbed oxygen on strained Pt overlayers. A recent work has further utilized XES technique to reveal electronic structure evidence of the anion redox in Li_2-*x*_RuO_3_ (1≤ x ≤ 2) in lithium-ion batteries (Fig. 5e)^52^. The oxygen *K*-edge XES spectra exhibit three major features: one major peak assigned to nonbonding oxygen and the low- and high-energy shoulders corresponding to bonding and antibonding Ru-O bonds, respectively. When charging Li_2-*x*_RuO_3_ up to 4.2 V_Li_ and beyond, a spectral broadening of the oxygen nonbonding feature was observed, originating from increased (O–O) σ* antibonding states, which is indicative of the formation of oxygen–oxygen coupling upon charging in the high-voltage plateau of 4.2 V_Li_. Ru *L*_3_-edge HERFD-XAS spectra further support this observation with the increased intensity in the high-energy shoulder upon lithium deintercalation as a result of the increased O − O coupling, inducing (O − O) σ*-like states with π overlap with Ru d-manifolds. By contrast, such broadening in XES spectra was not observed in charged Li_1-*x*_CoO_2_ and Li_1-*x*_Ni_1/3_Mn_1/3_Co_1/3_O_2_ (Fig. 5f), indicating the absence of lattice oxygen–oxygen coupling for lack of strongly covalent Ru-O bonds. These experimental XES spectra provide electronic structure fingerprints of oxygen–oxygen coupling and direct evidence central to lattice oxygen redox (O^2−^/(O_2_)^n−^), in charged Li_2-*x*_RuO_3_ after Ru oxidation (Ru^4+^/Ru^5+^) upon first electron removal with lithium deintercalation.

Recently, from the experimental Fe *K*_β_ spectra of Fe0.5 (MOF-based Fe–N–C material) powder and its CL (catalyst layer, after electrode preparation step), Saveleva et al. clearly show a gradual increase of the *K*_β_ʹ peak intensity accompanied by a *K*_β_ mainline shift toward higher emission energies with the increase of Nafion content (Fig. 5g)^53^. Such changes indicate a weak mixing between ligand and metal iron orbitals, that is, a weak metal–ligand covalency, which can be explained with a partial formation of octahedral sites (O_h_) from square-planar (D_4*h*_) configuration during the CL preparation as evidenced by Fe *K*_β_ HERFD-XANES spectra. Intriguingly, the valence-to-core XES spectra are further utilized to monitor the formation of iron oxides during Fe–N–C-based CL preparation. As shown in Fig. 5h, both iron oxide references present the low-energy valence-to-core feature around 7091 eV, *K*_β_ʹʹ, arising from Fe–O bond. However, such feature was not observed in the spectra of the Fe–N–C CL, validating no significant amounts of iron oxides during electrode preparation.

For electrochemical reactions, the adsorption configuration and selective interaction between reactant/intermediate with catalyst is in the foreground, however, such key information has been scarcely supported by solid experimental evidence so far. Researchers have recently utilized in situ valence-to-core XES to undoubtedly showcase the interactions with CO_2_ in monoclinic La_2_O_2_CO_3_ material under operando conditions^54^. By collecting emission spectra with an excitation in absorbance maximum, the nature of highest occupied states can be therefore probed. In Fig. 5i, in situ valence-to-core XES spectra display an intensity increase at −40 eV and an intensity decrease at −10 eV after exposing to CO_2_ at 250 °C, indicative of the direct adsorption of CO_2_ at La sites. The XES analysis is complemented by in situ HERFD-XAS to provide a holistic interpretation of interaction configuration, in which the observed changes in white-line intensity are ascribed to the presence of additional oxygen in the vicinity of La. Thus, it can be concluded that CO_2_ adsorbs at La_2_O_2_CO_3_ as surface carbonates, as schematically depicted in inset of Fig. 5i. Furthermore, the valence-to-core XES method has been recently applied to understand the atomic configuration of a CO_2_-reducing catalyst, despite not under operando conditions^55^. With a combination of valence-to-core XES and theoretical calculations, it identifies H_2_O/OH ligands bound to atomically dispersed iron sites on nitrogen-doped carbon toward CO_2_RR. Besides, titanium, chromium, manganese and iron complexes with different coordinated atoms (N, O, Cl, F) and alkyl ligands can be well distinguished by the valence-to-core XES^45,56–58^, which are not accessible by the conventional EXAFS method. This type of information obtained under working conditions would effectively decipher the binding mode of critical intermediates on catalytic sites and understand the realistic configuration at the molecular scale. Thus, we foresee that in situ XES approach could be quite powerful for determining the dynamic configuration of catalysts with complex interactions at the solid–liquid interface.

### Resonant inelastic X-ray scattering (RIXS)

As compared with the detection scheme in HERFD-XAS (energy scan for incident X-ray) and nonresonant XES (energy scan for fluorescence emission), in resonant inelastic X-ray scattering (RIXS), it performs a combination of energy scan for both incident radiation source and emitted fluorescence, which allows one to validate the energy difference (i.e., energy loss) between the initial state and final state via a coherent absorption-emission process (Fig. 2b). Accordingly, the RIXS technique combines the aspects of simultaneously probing unoccupied and occupied states of target elements, providing an element-dependent electronic excitation fingerprint. Significantly, if the energy transfer (i.e., inelastic scattering) is reduced to a range of a few electron volts (eV), RIXS spectroscopy, especially near the metal *L*-edge, can validate the relatively small energy transition in frontier orbital (i.e., *d–d* excitation), namely, a core electron excited to an empty *d* orbital (initial state) and the subsequent radiative decay of occupied *d* electrons to the core hole (final state), which is unlikely to be achieved through the other X-ray spectroscopies^59^. Such electronic excitations within the valence shell are highly sensitive to the chemical environment and coordinated ligand around the absorbing atoms, thus especially relevant for the bonding properties of the metal with adsorbates during reactions. Notably, although RIXS presents a close analogy to UV–vis spectroscopy in which UV and visible light can be used to excite 3*d* electrons to empty 3*d* states, in probing *d–d* transitions for transition metals, the high X-ray photons energy and two-photon selection process in the former make optically forbidden transitions (in UV–vis) accessible in RIXS, enabling a more reliable understanding of ligand field^59^. Another advantage of RIXS is to use hard X-ray irradiation for obtaining insightful information that is commonly achieved through soft X-ray experiments. For instance, 1*s*2*p* RIXS employing hard X-ray irradiation can be utilized to acquire *L*_2,3_-edge-like spectra of 3*d* transition metals^22,60^. Since the RIXS method involves both absorption and photoemission processes and further includes information on radiative decay, the spectrum measured via the second-order RIXS offers more informative features relative to that measured by the first-order *L*_2,3_ XAS. The utilization of hard X-ray sources in RIXS makes it more powerful for investigations of the solid–liquid interface under ambient conditions. In contrast, both the conventional UV–vis spectroscopy and soft X-ray XAS techniques are incompetent because the former one is difficult to deal with electrocatalytic solid–liquid interface and the latter case commonly requires an ultra-high vacuum (UHV) chamber and delicate measurement. Hard X-ray RIXS evidently provides those significant features about ligand bonding state and coordinated configuration of target elements, especially for the *d*-band configuration of transition metals during electrocatalysis^59^. Even if a major concern for RIXS technique is its weak spectroscopic features, because of rapid development of new-generation synchrotron facilities and a well-designed detection system, we anticipate that the element-specific and *d*-band sensitive RIXS technique holds great promise for deciphering transition-metal-mediated electrocatalysis.

**Fig. 6:**
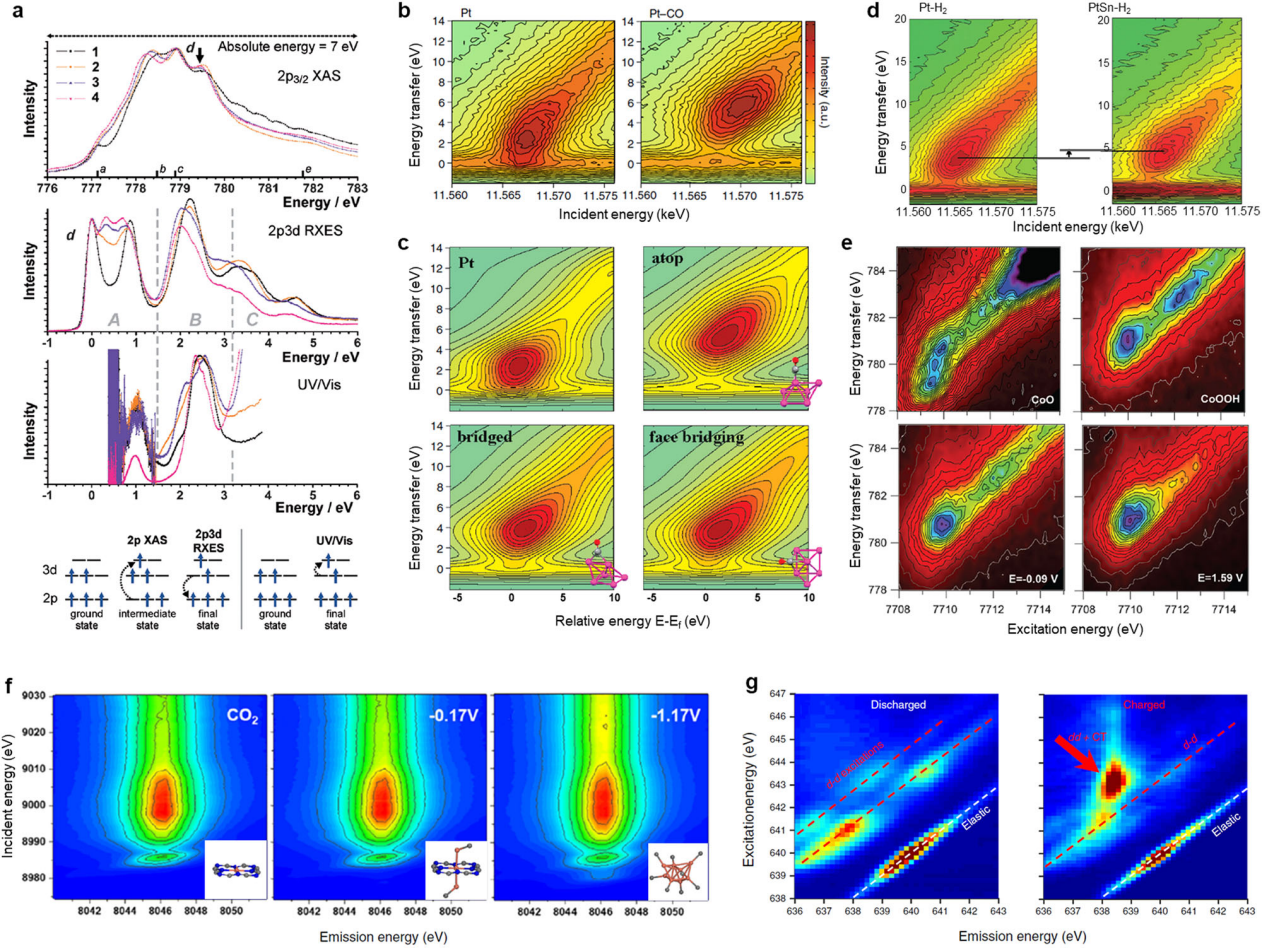
Recent progress in RIXS method on analyzing electrocatalysts and battery materials. **a** Experimental comparison of the sensitivity of XAS, RIXS, and UV–vis techniques to *d–d* excitations in cobalt carboxylates. From top to bottom panel: Co 2*p*_3/2_ XAS spectra, 2*p*3*d* RIXS spectra at excitation energy d, UV–vis spectra of 1–4 compounds, and schematic representations of the photon-induced electron transitions in 2*p* XAS, 2*p*3*d* RIXS and UV–vis spectroscopy. The final states in 2*p*3*d* RIXS and UV–vis spectroscopy are identical. The numbers a-e indicate five energies at which spectra were acquired. **b** 2*p*_3/2_-5*d* RIXS planes of supported Pt nanoparticles: (left) metallic and (right) with CO adsorbed. **c** Calculated RIXS planes for a bare Pt_6_ cluster and the cluster with CO coordinated at three different sites. **d** 2*p*_3/2_-5*d* RIXS planes of (left) Pt/Al_2_O_3_ and (right) PtSn/Al_2_O_3_ after hydrogen adsorption. **e** In situ RIXS planes of the Co oxide/Au(111) electrocatalyst at various potentials during OER, along with the RIXS planes of CoO and CoOOH standards. **f** Operando *K*α_1_ RIXS planes of CuPc measured at various potentials in 0.5 M KHCO_3_ solution during CO_2_RR. **g** Mn *L*_3_-edge RIXS maps on MnHCMn electrodes at the discharged (left panel) and charged (right panel) states in Na-ion battery, respectively. Figures adapted with permission from: **a** ref. ^59^, Wiley (2013); **b**, **c** ref. ^61^, American Chemical Society (2010); **d** ref. ^62^, RSC (2010); **e** ref. ^33^, RSC (2013); **f** ref. ^63^, Elsevier (2022); **g** ref. ^65^, Springer Nature Ltd (2018).

In a recent work, an experimental comparison on the ligand field sensitivity among different spectroscopies for a series of cobalt(II) carboxylates has been conducted. Spectral features reveal the highly element-dependent sensitivity of 2*p*3*d* RIXS method to *d–d* excitations as compared with UV–vis, while 2*p* XAS cannot probe such electronic excitations within the *d* band (Fig. 6a)^59^. Ligand field multiplet calculations further validate that 2*p*3*d* RIXS allows the most judicious identification of ligand field parameters, which can precisely determine the crystal field and coordination configuration around metal ions. It is well known that, for most catalytic process, the nature of frontier orbitals that is strongly dependent on the interaction between the *d* band of transition-metal sites and *p* band of adsorbates determines catalytic fate, which has to be unraveled by advanced experimental technologies. Significantly, the RIXS method is considerably powerful in clarifying such electronic structure changes around the frontier orbitals of the catalytically reactive sites. Figure 6b displays the RIXS planes of alumina-supported Pt nanoparticles with and without CO adsorption, which shows the charge transfer distribution that is representative of occupied *d* densities of states (DOS)^61^. For bare Pt nanoparticles, the elastic peak (at zero energy transfer) was observed to merge with the valence-band excitations, indicating that the Fermi level lies within a partially filled band. Upon CO adsorption, the energy distribution broadens, and a gap opens up between the elastic peak and the occupied states, which suggests the downshift of 5*d* valence band originating from the orbital hybridization between *d* state of Pt and *s-p* state of the ligand. Interestingly, based on theoretical analysis, it is confirmed that the CO predominantly adsorbs on the platinum surface in an atop configuration (Fig. 6c). Hard X-ray RIXS has been further employed to study the *d*-DOS of Pt and PtSn alloy nanoparticles upon reactants adsorption of H_2_ and CO^62^. Analysis on the RIXS planes shows a downward shift and a narrowing of *d* band in Pt relative to the Fermi level after alloying with tin. After adsorbing H_2_, the *d* band in PtSn becomes narrow along the incident energy, and the center of the most intensive feature shifts toward high position as compared with Pt sample, validating the further downward shift of *d* band in PtSn, which indicates the unfavorable H_2_ adsorption (Fig. 6d).

Recently, renewable energy research has found its way to RIXS technique for investigating electrocatalytic processes. By using advanced operando RIXS with hard X-ray irradiation, the oxidation states of Co sites in various references and electrodeposited CoO_x_ catalyst during OER have been clearly discriminated. It can be seen that the RIXS planes of the pre-edge region in CoO_x_ catalyst exhibit similar nature with that of CoOOH reference over a wide potential range (Fig. 6e)^33^. However, the non-local transition to 3*d* states observed at the excitation energy of 7713 eV in the CoOOH spectrum is notably weaker in the CoO_x_ spectra over the OER course. Another excellent example is the detection of dynamic configuration changes of CuPc electrocatalyst under CO_2_RR conditions by RIXS^63^. As shown in Fig. 6f, operando *K*α_1_-RIXS planes demonstrate that a new transition peak at incident energy of 8980 eV appears at −0.17 V versus RHE, and is gradually strengthening as the applied potential increases, indicating the presence of a unique structure in the catalysts. From the RIXS plane, HERFD-XANES spectra that are obtained by integrating the profile along the constant emission energy (8046.3 eV) provide exhaustive electronic and geometric information, which further confirms the dynamic reconstruction from single atoms into potential-induced copper clusters in a CuPc catalyst during the CO_2_RR process. Notably, HERFD-XANES spectrum is essentially a cross section from the RIXS plane^13^, any features off from the cross section will be missed in this single spectrum. Taking Fe species as an example, pre-edge features are in fact strongly dependent on valence and spin state, which exhibit multiplet nature, however, some of these lie off from the cross section and thus cannot be well reflected in HERFD-XANES spectra^64^. These findings indicate the superior possibilities of RIXS to provide more detailed information on the electronic structure as compared with XAS. Furthermore, because different numbers of 3*d* electrons substantially lead to variable *d*–*d* excitations, RIXS can be utilized as a sensitive probe for distinguishing the Mn^1+^ and Mn^2+^ of manganese(I/II) anode in sodium-ion battery^65^. As shown in Fig. 6g, the discharged (Mn^2+^) sample exhibits several *d*–*d* excitation features that are typical for a Mn^2+^ system with partially occupied t_2g_ and e_g_ states. In contrast, the charged sample displays a greatly enhanced RIXS feature with a relatively high-energy-loss value (away from the elastic line for about 5 eV) at the ~643.4 eV excitation energy, which directly identifies the low-spin 3*d*^6^ configuration with fully occupied t_2g_ states, i.e., Mn^1+^, at the charged states.

### Time-resolved X-ray spectroscopies

As discussed above, those advanced X-ray spectroscopies are emerging to achieve promising energy-resolved spectral features, which significantly empowers the atomic- and molecular-level understanding of configurations at the electrochemical solid–liquid interface. To consider the practical situation of electrocatalysis, dynamic behaviors, and transitions take place transiently at the interface, for instance, the dynamic reconstruction and phase transformation in electrocatalysts can rapidly occur within a few minutes or seconds^4,66^. However, it is noted that the acquisition of a full XAS spectrum in a conventional way generally needs several 10 min, and particularly requires much longer time for spectra acquisition in those advanced X-ray spectroscopy experiments such as HERFD-XAS, XES and RIXS, which cannot fulfill the time scales required for tracking the dynamic electrocatalytic behaviors. This means that these techniques just provide “steady state” information on the rate-limiting condition rather than “transition state” understanding of dynamic events under working conditions. As a consequence, time-resolved X-ray spectroscopies that can realize real-time monitoring of dynamic transformations and shed light on the yet unknown details of those transition steps is extremely paramount, especially for in situ/operando studies. For XAS method, note that the poor temporal resolution can be attributed to a slow energy scan which is restricted to its mechanical scanning of monochromators for incident X-ray source as operated in both XAS and HERFD-XAS techniques^67^. In this regard, a quick-scan monochromator has been developed for achieving time-resolved X-ray absorption spectroscopy, which enables rapid and continuous energy scans by smoothly oscillating the monochromator crystals^68^. Nowadays the state-of-the-art quick-scan monochromator can realize a temporal resolution of 2 ms^69^.

**Fig. 7:**
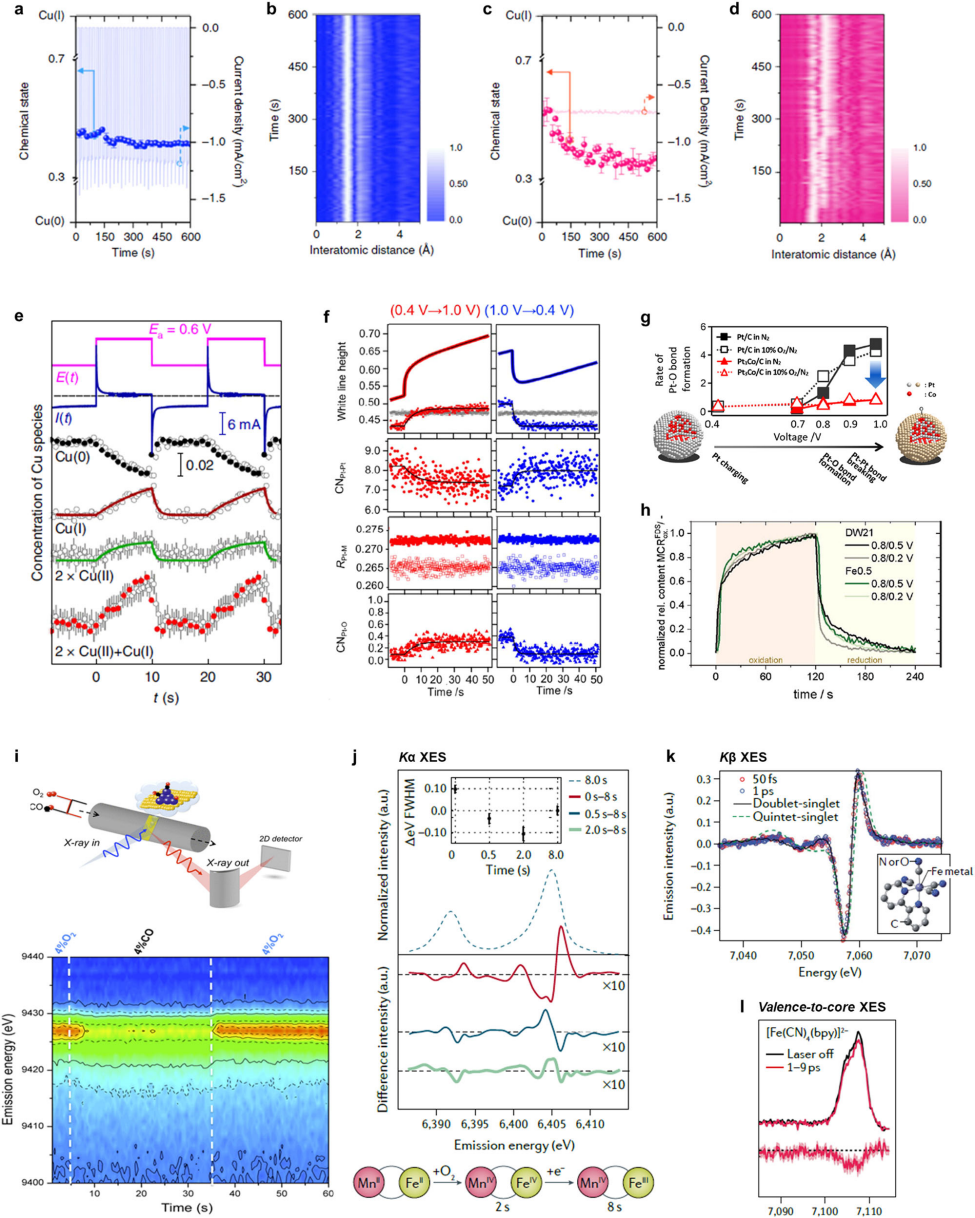
Advanced time-resolved X-ray spectroscopies for energy-conversion processes. **a**, **c** Time-resolved variations of Cu species in CuO_x_ by using (**a**) the potential switching method and (**c**) the chronoamperometry method, along with corresponding electrochemical responses during CO_2_RR at −0.75 V versus RHE. **b**, **d** Corresponding time-dependent EXAFS spectra during CO_2_RR from a top view. **e** Concentration evolutions of Cu^0^, Cu^+^, and Cu^2+^ species in Cu_2_O nanocubes under pulsed conditions, extracted from EXAFS data fitting (filled black and red circles) and LCA-XANES analysis (gray open circles). **f** Time profiles of structural parameters of Pt_3_Co/C for the voltage cycling processes in N_2_ cathode flow. **g** Time-dependent rate constants of Pt–O bonds in Pt/C and Pt_3_Co/C during the transient voltage operation with the exchange of the cathode gas, along with a schematic of the kinetics of cathode surface events from 0.4 to 1.0 V versus RHE. **h** Time-dependent concentration profiles of the oxidized component for both DW21 and Fe0.5 single-atom catalysts with the potential switching to 0.5 and 0.2 V versus RHE. **i** Top: schematic representation of in situ time-resolved high-energy-resolution off-resonant spectroscopy (HEROS) using the von Hamos-type spectrometer. Bottom: temporal evolution of the HEROS spectra on Pt/Al_2_O_3_ during CO/O_2_ switches at 300 °C. **j** Fe *K*α XES spectra of solutions of Fe/Mn containing ribonucleotide reductase R2c at 25 °C for various O_2_ exposure times. The inset shows the *K*α_1_ FWHM as a function of exposure time relative to an 8-s exposure. **k** Transient Fe *K*β XES difference spectra of Fe(CN)_4_(bpy) (bpy = 2,2’-bipyridine; structure in inset) at 50 fs and 1 ps after photoexcitation and the calculated difference. **l** Transient Fe valence-to-core XES spectra of Fe(CN)_4_(bpy). Figures adapted with permission from: **a**–**d** ref. ^67^, Springer Nature Ltd (2020); **e** ref. ^70^, Springer Nature Ltd (2022); **f** ref. ^71^, American Chemical Society (2014); **g** ref. ^72^, American Chemical Society (2018); **h** ref. ^74^, Wiley (2022); **i** ref. ^76^, American Chemical Society (2013); **j** ref. ^78^, Springer Nature Ltd (2017); **k** ref. ^79^, RSC (2017); **l** ref. ^80^, AIP Publishing (2020).

To determine actually reactive species for electrocatalytic reaction, the only truly reliable option is to perform operando transient X-ray spectroscopies instead of pseudo stead state measurements. Recently, the quick-XAS with a time resolution of several seconds has been employed for realizing the seconds-timescale studies on the Cu oxide surface under CO_2_RR conditions, which has delivered the snapshots of ultrafast chemical state changes in copper-based catalysts during CO_2_RR^67^. As shown in Fig. 7a–d, it clearly reveals that the employment of a potential switching approach for CO_2_ electroreduction can balance the chemical state of half-Cu^0^-and-half-Cu^+^ on catalyst surface, while the conventional chronoamperometry method drastically reduces Cu oxide to metallic states. The coexisting Cu^0^-Cu^+^ ensembles experimentally observed on the former catalyst surface are clarified to result in a selective production of ethanol on Cu-based systems. Furthermore, via operando XAS experiments in quick XAFS mode with subsecond time resolution, the periodic changes of catalyst structure and composition in Cu_2_O under pulsed reaction conditions have been deeply tracked^70^. Quantitative results demonstrate that the Cu^0^ faction decreases upon applying anodic pulse and increases during the cathodic pulse, while opposite trends were observed in the Cu^+^ and Cu^2+^ amounts (Fig. 7e). The oxidation and reduction processes were observed to be highly asymmetric, with the latter being much kinetically faster, suggesting that Cu oxides with mixed chemical state (Cu^+^/Cu^2+^) only formed in the near-surface layers of the catalyst, while the core is still metallic during the anodic pulse. By linking the time-dependent concentration map of Cu oxide species with the selectivity profiles, it can be inferred that a twofold increase in CO_2_-to-ethanol conversion is ascribed to an optimized dynamic balance between distorted Cu oxides and metallic Cu species rather than the irreversible morphological evolution in a narrow range of pulse durations. Such quick-XAS with the time resolution in the 100 ms-range has also been instrumental for investigating the structural kinetics of Pt cathode catalysts under polymer electrolyte fuel cell operating conditions, as demonstrated by some pioneering studies^71–73^. Operando time-resolved Pt *L*_3_-edge XANES and EXAFS spectra for instant voltage operations in cathode gas flow provide the time profiles of structural parameters of Pt catalysts and the rate constants of various structural transformations (Fig. 7f)^71^. It is observed that a slower reaction rate of Pt-Pt bond re-forming than that of Pt–O bond breaking significantly affects the redox processes of Pt, leading to a catalyst degradation during power-on/power-off cycles in PEMFC. Meanwhile, operando quick XAFS measurement also validates that an alloying of 3*d* transition metals with Pt kinetically improves the reduction of Pt from 1.0 to 0.4 V versus RHE, while the rate enhancement of reverse Pt oxidation process is considerably negligible, which explicates the improvements of activity and durability of Pt catalysts with Co or Ni alloying. In particular, considering the rapid exchange of cathode gas (N_2_ → 10% O_2_ in N_2_) under PEMFC operations, operando time-resolved XAS analysis has evidenced the consistency in structural kinetics of Pt-based catalysts in N_2_ and in 10% O_2_/N_2_ (Fig. 7g)^72^. Recently, in addition to Pt cathodes, researchers have innovatively employed the quick-scan XAS with a time resolution of 2 s for in situ study of transformation kinetics in Fe/N/C single-atom catalysts during ORR^74^. As illustrated in Fig. 7h, the rate of both oxidation and reduction processes are similar for Fe0.5 sample, while the oxidative process proceeds significantly slower than the reductive process did for DW21 sample. The sluggish oxidation kinetics in the formation of O-based surface adsorbates for DW21 sample could be referred to its ~30-fold lower oxygen reduction activity as compared with Fe0.5 sample.

Significantly, to probe the spin and electronic interaction dynamics of metal sites on ultrafast time scales, one has to rely on the development of time-resolved XES approach. In the time-resolved XES measurement, instead of a scanning monochromator, an energy-dispersive polychromator (such as an analyzer in von Hamos geometry using a cylindrically curved crystal to produce a polychromatic line focus) is frequently implemented^75^. White X-rays are poly-chromatized to angle-resolved X-rays, which pass through the sample and expand as energy-dispersive X-rays that are collected by a position-sensitive detector. Because of the scanning-free arrangement and simultaneous record of all parts of the spectrum (“single-shot” experiment), the XES data can be measured with very high time resolution. For instance, a typical work has used this approach to monitor the dynamic oxidation and reduction steps of Pt catalysts during CO oxidation with subsecond time resolution (Fig. 7i)^76^. Since these XES spectra are collected in a single shot, the time resolution depends only on the number of incoming photons and element concentration, which thus can be further improved particularly by the X-ray free-electron lasers (XFEL)^77^. XFEL-based XES has been utilized to investigate changes in oxidation state and spin state of metals atoms in enzyme systems (Fig. 7j)^78^. Time-dependent Fe *K*α XES spectra clearly reveal the formation of a Fe^IV^ intermediate (within ~2 s), followed by the catalytically active Fe^III^ state. Recently, picosecond-resolved Fe *K*β XES spectra based on XFEL have been realized and monitored the initial steps of photoexcitation and light-induced charge transfer of photosensitive transition-metal complexes (Fig. 7k)^79^. Transient XES spectra evidence that only one excited state, a doublet metal–ligand charge transfer, is present after photoexcitation. Furthermore, the valence-to-core XES is also extended to ultrafast time-resolved experiments for probing changes in geometric and electronic structures induced by photoexcitation in the femtosecond time domain using an XFEL. As shown in Fig. 7l, transient Fe valence-to-core XES spectra display an intensity decrease and a blue energy shift, indicative of the increasing metal–ligand distance and the photo-oxidation, respectively^80^. Note that, the XFEL-based time-resolved XES spectroscopy has been only applied in repeatable processes so far, such as photocarrier lifetimes and charge transfers of catalytic species. Although no advance has been reported regarding this technique on electrocatalysis, it indeed paves a way for making extensive use of hard X-ray spectroscopies in ultrafast studies, which is particularly useful in revealing the elementary steps of electrocatalysis.

## A comprehensive picture of the electrochemical solid–liquid interface

Above-mentioned advanced X-ray spectroscopies do provide unprecedented opportunities to reveal important evidence on the atomic configuration for interfacial electrocatalysis. However, to further achieve comprehensive insights into the molecular-level solid–liquid interface toward target reactions, significant complementarities from the following aspects in terms of experimental manner is strongly suggested to be considered.

### Complementary soft X-ray spectroscopy

X-ray absorption spectroscopy for *K*-edge transition (1 *s* → n*p*) is commonly utilized to reveal the locally structural information (i.e., interatomic distance as well as coordination number) and the electronic situation. However, n*p* orbitals are relatively insensitive to the catalytic reaction as compared with (n-1)*d* orbitals because of a fact that, in transition metals, the frontier orbitals are mainly contributed by *d* orbitals while only few parts are characteristic of *p* orbital. Toward this end, *L*-edge transition (2*p* → (n – 1)*d*) can acquire the electronic structure of the unoccupied (n– 1)*d* orbitals that are really involving the chemical reaction and catalytic nature of a transition metal, which directly observes the chemical bonding between the reactive metal centers and the adsorbates during catalytic reactions. For instance, most of the interesting catalysts are first-row transition metals, which requires a *L*-edge transition from 2*p* to 3*d* (i.e., in a soft X-ray region (<1 KeV)). Notably, owing to the extremely short mean free path of soft X-ray in the ambient or liquid environment, most soft X-ray experiments have to be conducted in UHV conditions. To conquer such experimental difficulty, many studies have demonstrated some UHV in situ cells, in which a silicon nitrate film acts as a separator to protect the UHV condition from aqueous reaction system and the signals are collected from the substrate side rather than the side that proceeds the catalysis^81,82^.

**Fig. 8:**
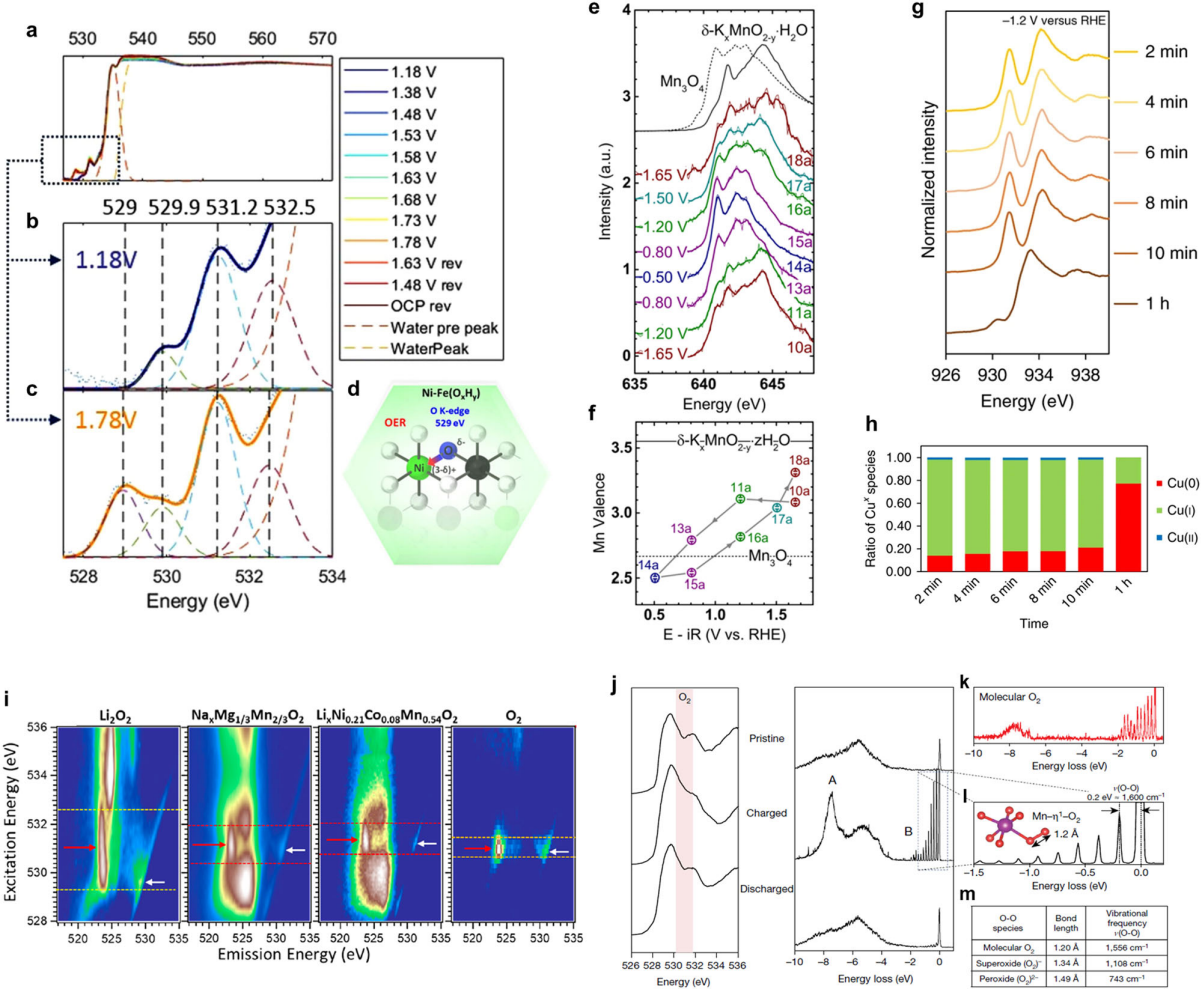
Complementary soft X-ray XAS spectroscopies. **a** In situ soft X-ray XAS spectra at the O *K*-edge of electrodeposited Ni–Fe electrocatalyst during alkaline OER. **b** O *K*-edge prefeature region at 1.18 V versus RHE. **c** O *K*-edge prefeature region at 1.78 V versus RHE. **d** Schematic illustration showing a partial donation of the higher electron density on the oxygen site (O^δ−^) to the Ni metal site (O → Ni charge transfer). **e** In situ soft X-ray XAS spectra at the Mn *L*_3,2_-edges of the electrodeposited manganese oxide recorded in inverse partial fluorescence mode. **f** Trends of the Mn valence during electrochemical cycling. **g** In situ soft X-ray XAS spectra at the Cu *L*_3_-edge of the electro-redeposited copper catalyst at −1.2 V versus RHE for 1 h CO_2_RR electrocatalysis. **h** The ratio of Cu species with time during 1 h of CO_2_RR at −1.2 V versus RHE. The data are extracted from the linear combination analysis of soft X-ray XAS spectra. **i** Mapping of RIXS profile of oxidized oxygen states in Li_2_O_2_, charged Na_2/3_Mg_1/3_Mn_2/3_O_2_, charged Li_1.17_Ni_0.21_Co_0.08_Mn_0.54_O_2_, and O_2_. **j** Oxygen *K*-edge XAS and high-resolution RIXS spectra recorded at an excitation energy of 531 eV for honeycomb-ordered Na_0.75_[Li_0.25_Mn_0.75_]O_2_ in the pristine, charged (4.5 V), and discharged (2 V) states. The red highlighted pre-edge feature at 531 eV and RIXS features A and B are characteristic of O-redox. **k** The high-resolution RIXS spectrum for molecular O_2_ at 530.3 eV. **l** With high-resolution RIXS, feature B in (**j**) is resolved into a progression of energy-loss peaks, arising from the vibrations of the O–O bond with a vibrational frequency of 1600 cm^−1^ matching that of molecular O_2_ and that expected from the 1.2-Å O–O bond in the Mn–η^1^–O_2_ species predicted from DFT. **m** Literature values for the bond lengths and frequencies of O–O dimers for comparison. Figures adapted with permission from: **a**–**d** ref. ^83^, Springer Nature Ltd (2019); **e**, **f** ref. ^89^, American Chemical Society (2017); **g**, **h** ref. ^90^, Springer Nature Ltd (2018); **i** ref. ^95^, American Chemical Society (2020); **j**–**m** ref. ^96^, Springer Nature Ltd (2020).

Soft X-ray spectroscopy is a primary tool to study those light elements (e.g., C, N, O) in electrocatalysts since their *K*-edge ionization energy lies approximately between 200 and 1000 eV. These elements are frequently involved in reactant, intermediate, and product species, thus in situ probing those elements can provide critical insights about the dynamic structure of catalyst^83^, or those of the electrolyte^84^ and catalyst-adsorbate interactions^85^. For instance, the local bonding states and symmetry characteristics of oxygen atoms in an electrodeposited Ni–Fe(O_x_H_y_) electrocatalyst for alkaline OER are studied by in situ XAS of oxygen *K*-edge (Fig. 8a–d)^83^. The oxygen *K*-edge spectra include two main regions: the low-energy region at 525–534 eV represents the electronic transition from O(1 *s*) to O(2*p*) hybridized with M(3*d*); the energy region at 534–540 eV is the near edge feature of the water molecule. Four peaks at 529, 529.9, 531.2, and 532.5 eV are distinguished as the transitions from O(1 *s*) to O(2*p*) hybridized with Ni(3*d*)t_2g_, Fe(3*d*)t_2g_, O(π*) of O_2_, and Fe(3*d*)e_g_, respectively. With increasing applied potentials, a new pre-peak feature at 529 eV was observed to intensify at 1.48 V versus RHE and disappear reversibly at cathodic potentials, coinciding with the Ni^2+^/Ni^3+^ redox. This phenomenon can be explained by an increase in hybridization between Ni(3*d*) and O(2*p*), which causes electron redistribution between O and Ni sites and improves the OER activity. On the contrary, the peaks at 529.9 and 532.5 eV that are assigned to the transitions from O(1 *s*) to O(2*p*) hybridized with Fe(3*d*) show slight changes with increasing applied potentials, suggesting that Fe sites undergo a small modulation as compared to Ni sites. In situ oxygen *K*-edge results thus unravel the orbital hybridization of O(2*p*) with metal ions and charge transfer from O(2*p*) to metal ions, which well explicates the dynamic evolution during electrocatalysis. Moreover, thanks to the energy difference observed for carbon species^86^, the ability to probe the oxidation state of carbon through carbon *K*-edges XAS allows to discriminate among CO_2_, CO, and more reduced forms of carbon, which is expected to provide valuable information on the critical intermediates, binding mode as well as their implication in non-covalent bonds in the CO_2_RR and beyond. In a typical work^87^, the adsorption of CO_2_ on CeO_2_(110) has been investigated with carbon *K*-edge XANES, which identifies two different adsorbates, i.e., CO_3_^2−^ and CO_2_, depending on the nature of the surface. Another study also employs carbon *K*-edge XANES to demonstrate that there is a stronger CO binding on deep-pitted (low coordinated) Au(111) sites than that of flat surfaces^88^, which is consistent with the results of infrared (IR) reflection spectra, showing the great potential of soft X-ray XAS techniques in identifying interfacial light elements that are participating in the formation of chemical bonding.

More significantly, soft X-ray XAS techniques allow to probe the *L*-edges of 3*d* transition metals, which provide direct information on the oxidation state and geometric structure of the absorbing atom as compared with *K*-edges. Recently, the dynamic oxidation states in an electrodeposited manganese oxide film at OER- and ORR-relevant potentials have been evidenced with Mn *L*_3,2_-edge XAS spectra (Fig. 8e)^89^. At potentials above 1.50 V versus RHE (steps 10a, 17a, and 18a), the spectra match well with that of the δ-K_x_Mn^3.5+^O_2-y_·zH_2_O reference, while at potentials below 0.80 V versus RHE (steps 13a, 14a, and 15a) the spectra are consistent with that of the Mn^2.7+^_3_O_4_ reference containing Mn^2+^ at tetrahedral site. These findings suggest that the manganese oxide contains tetrahedral Mn^2+^ site during ORR, while it is characteristic of a mixed Mn^3+/4+^ valence during OER. Based on Mn redox behaviors during cycling the potentials (Fig. 8f), one can further deduce that the oxidation kinetics of the manganese oxide is more sluggish than the reduction kinetics. Moreover, note that soft X-ray XAS is considered surface-sensitive as compared to hard X-ray case, the dynamic surface oxidation state of an electro-redeposited Cu oxides during CO_2_RR can be probed by in situ Cu *L*-edge XAS measurement as well (Fig. 8g)^90^. Linear combination analysis on in situ Cu *L*-edge XAS spectra shows that surface Cu species are made of 84% Cu^+^ in the initial 2 min electrocatalysis at −1.2 V versus RHE, then the Cu^+^ amount decreases to 77% in the following 10 min. After 1 h CO_2_RR electrocatalysis, 23% of Cu^+^ species are still remained (Fig. 8h), which is suggested to be responsible for the efficient CO_2_-to-ethylene conversion. In addition, detailed shape and intensity analysis of *L*-edges can also reveal the structure of the adsorption sites that are involved in bonding to reactants and intermediates^91,92^, such as ethene adsorption sites on supported metal catalysts.

Intriguingly, because the soft X-ray region covers the 1 *s* to 2*p* excitations of light elements (e.g., C, N, O, Li) and 2*p* to 3*d* transitions of first-row transition metals, in situ soft X-ray techniques exhibit powerful capabilities in tracking the dynamic interfacial behaviors in lithium-ion battery and beyond. Excellent reviews on the in situ soft X-ray spectroscopies for rechargeable batteries have been recently published by Lin et al. and Yang et al.^93,94^. Moreover, soft X-ray RIXS with the capability to distinguish the same element with inequivalent chemical environment is ideal to monitor electronic structure in various battery systems. For instance, the oxygen redox states are investigated by collecting the oxygen *K*-edge mapping of RIXS from O_2_, Li_2_O_2_, and two representative Na/Li-ion battery electrodes at charged states with oxidized oxygen (Fig. 8i)^95^. The direct comparison of RIXS profiles displays that, although all these oxidized oxygen species show the critical feature around the 523.7 eV emission energy (red arrows) and an enhanced low-energy excitation feature close to the elastic line (white arrow), those features display different distributions along excitation energies for different systems. Especially, the contrast in widths and positions of the characteristic 523.7 eV emission feature strongly suggests that the oxidized oxygen state involved in oxygen redox reactions in batteries is not simply through a pure molecular configuration of either a peroxide type or oxygen gas. These observations indicate the strong association between the transition metal and oxygen in batteries, it is thus suggested to go beyond both a molecular oxygen configuration and the hybridization model to truly understand the oxygen redox activities in a complex transition-metal oxide electrode. In another recent work, the oxidized oxygen in cathodes is probed by using a high-resolution soft X-ray RIXS spectroscopy^96^, which reveals the underlying fine structure of the elastic peak (labeled B in Fig. 8j), indicating a progression of energy-loss peaks associated with the vibrations of O–O bond matching with that of the molecular O_2_ (Fig. 8k–m). The RIXS observations strongly support the molecular O_2_ with η^1^ coordination to Mn site during charging. By contrast, both spectroscopic features A and B disappear in the discharged sample, indicating that the O_2_ species is reduced during discharging. Thus, the soft X-ray RIXS presents high sensitivity for the subtle changes and enables the discovery of the buried features in the pre-edge of oxygen *K*-edge XAS spectra.

### Complementarity among emerging X-ray spectroscopies

**Fig. 9:**
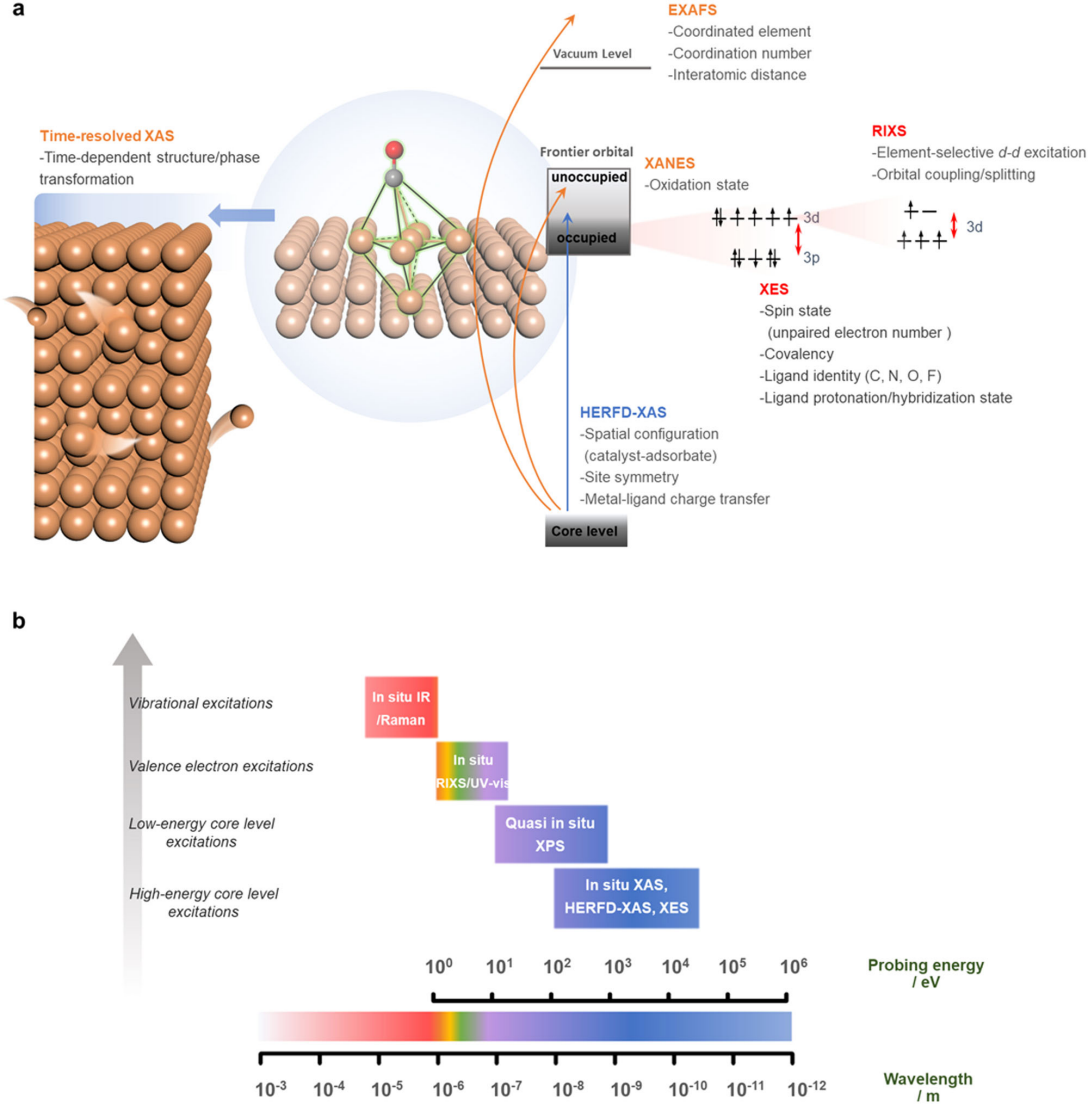
Complementarity among advanced X-ray spectroscopies. **a** Schematic illustrating complementarities of X-ray spectroscopies in deciphering electrocatalysis at the solid–liquid interface. **b** Probing energy-dependent “in situ spectroscopy map” for comprehensively understanding electrocatalysis at the interface in a complementary way.

The complementarity among the advanced X-ray spectroscopies and conventional XAS is illustrated in Fig. 9a. As one of the most widely used X-ray techniques for electrocatalytic studies, XAS method provides insightful information on the dynamic natures of electrocatalysts. XANES validates the nature that core electrons are excited by an incident irradiation to corresponding unoccupied states, it is thus strongly sensitive to oxidation state (density of unoccupied states) of the absorbing atom. In contrast, EXAFS is capable of providing that quantitatively structural information, such as interatomic distances and coordination numbers around the metal site. Nevertheless, it has to be noticed that those chemical bonds between absorbing atoms (i.e., transition metals) and reactant/intermediates during reactions are mostly involving hybridized *d* orbitals of reactive centers rather than their unoccupied *p*-character in frontier orbitals. Such hybridized *d* orbitals around reactive metals strongly correlate with the local symmetry of absorbing site, therefore the spectral features in pre-edge region can hint the symmetrical configuration of site–adsorbate interactions. Because of the dipole-selection rule for forbidden transition from *1* *s* to (n – 1)*d* state for *K*-edge transition, the interested character of hybridized *d* states in transition metals show extremely weak features in XANES (pre-edge region). Owing to the limited energy resolution resulting from the intrinsic lifetime broadening, pre-edge features cannot be well identified in conventional XAS mode. Toward this end, high-energy-resolution XANES features can be achieved by the so-called HERFD-XAS technique that allows to efficiently extract insightful information based on sharpened features. Consequently, as compared with conventional XAS method, HERFD-XAS is characteristic of complementary nature to validate the interestingly hybridized *d* states through the forbidden *1* *s* to (n – 1)*d* transition and further offers the insightful information regarding catalyst-adsorbate configuration, symmetry and metal–ligand charge transfer around the reactive centers.

By contrast, the occupied states of reactive centers containing highly complementary information can be realized by employing XES and RIXS approaches depending on the measuring configuration, whereas those unoccupied states can be described by XAS methods (i.e., XANES and HERFD-XANES). In XES manner, after electron holes at core level are created by nonresonant excitation, following by refills of electrons at higher energy levels, several emitted fluorescence lines can be generated for further analysis. For instance, the *K*_β_ mainline emission, 3*p* → 1 *s*, can be referred to an interaction between 3*p* and 3*d* orbitals. It can be attributed to a fact that presence of unpaired 3*d* electrons leads to significant spin state and further results in a strong spin-orbital coupling in 3*d* orbital, which causes significant interactions between 3*p* and 3*d* orbitals and makes the 3*p* orbital split. Accordingly, two *K*_β1,3_ and *K*_β_ʹ spectral features respectively corresponding to the ^7^P and ^5^P final states can be obtained because of the presence of unpaired electrons at valence shell of 3*d* transition metals and the 3*p* − 3*d* exchange coupling. That is, *K*_β_ mainline emission is sensitive to the spin state and metal–ligand covalency for 3*d* transition metals. On the other hand, the *K*_β_ lines furthermore show weak satellite features on their high-energy side, which arise from the fluorescence after the decay of an electron from the valence orbitals of 3*d* transition metals to refill the core hole, denoted as *K*_β2,5_ and *K*_β_ʺ, thus directly reflecting the configurations of electron orbitals that participate in the chemical bond. Especially, from *K*_β_ʺ peak, various coordinated light elements (e.g., C, N, O and F) or different protonated situation (e.g., H_2_O, OH^−^, and O^2−^) can exhibit considerable spectral changes, which have unique capabilities to identify the coordinated nature around the metal centers. As a consequence, as compared with XAS reflecting those unoccupied states, the XES is the approach to offer valuable information about occupied states that can refer to their unpaired electrons (i.e., spin state), bonding covalency as well as the identification of light elements around absorbing atoms.

Due to the selection rules for electric dipole transitions, XES shows a shortcoming in a poor understanding of *d* valence shell that really participates in interacting/bonding with reactants and/or intermediates, because none of the emission line for *d–d* transition can be validated^75^. Fortunately, RIXS can provide a promising solution to well resolve the *d* valence shell since the RIXS approach can be regarded as a combination of XAS and XES, which focuses on those energy differences between the incident irradiation and emitted fluorescence, namely the energy transfer (energy loss, Ω–ω). Such concept is similar to that of Raman spectroscopy which obtains the energy difference caused by inelastic scattering to reveal the small energy change as a result of molecular vibrations. Because the energy resolution of RIXS can achieve sub-electron volts (eVs), the *d* orbital splitting caused by crystal field effects and interactions with surround environments can be evidently validated in RIXS planes. Consequently, as compared to those of XAS and XES methods, RIXS can be a high-resolution approach toward the frontier orbitals. On the other hand, once the magnitude of energy transfer is approximately several tens of eVs, RIXS can also provide similar information on the electronic structure that is conventionally accessed by soft X-ray *L*- and *M*-edge spectroscopies, such as 1*s*2*p* and 1*s*3*p* RIXS^97^. These emerging hard X-ray spectroscopies that do not require vacuum system can act as a powerful and complementary toolbox to interrogate the interfacial configurations of electrocatalytic researches.

### A comprehensive probing energy-dependent “in situ spectroscopy map”

Despite above-mentioned powerful X-ray spectroscopies, it should be noted that those X-ray approaches generally provide informative features regarding the subsurface and partial near-surface of electrocatalysts and are characteristic of limited capabilities of directly tracking the dynamic adsorbate species and their configurations at the solid–liquid interface. Furthermore, as we mentioned above, the XAS may not provide qualified data to perform reliable fitting analysis. Consequently, a comprehensive understanding of the atomic configuration at interface under working conditions is hardly achieved through monomodal X-ray spectroscopy approach. For electrocatalysis that typically occurs at the solid–liquid interface with presence of complex interactions between electrocatalyst and varied environment, dynamic configurations at different landscape from interface, surface to bulk phase are considerably complicated, and thus it is pressing to combine several complementary in situ/operando techniques for deciphering a whole picture of dynamic solid–liquid interface.

As illustrated in Fig. 9b, we aim to highlight the complementarities among various in situ/operando techniques from a view point of probing energy scales. With the various probing energy, in situ/operando techniques that have their unique inherent resonance/interplay relating to specific dynamic event can be well understood and classified into following categories: (i) in situ Raman and Infrared (IR) spectroscopies, by the use of light energy that is responsible for vibrational excitation, such methods usually probe vibrational spectra in mid-infrared region. IR detects the specific absorption of molecular vibrations and is thus apt for monitoring adsorbed species on metallic electrode surfaces, while Raman collects the inelastically scattered light resulting from vibrational or rotational transitions of target substances with an incident monochromatic irradiation, thereby being especially for tracking the type and configuration of most organic species (e.g., intermediate species during reactions) as well as the chemical states on catalyst surface. More detailed complementarities between Raman and Infrared spectroscopies have been well explicated elsewhere^98^. (ii) In situ X-ray spectroscopies, they probe the inelastic scattering of X-ray with the energy ranging from a few eVs to thousands of eVs, which can cover the inherent resonance ranging from the electronic transition of inner electrons (core level) and the valence electrons in frontier orbitals. In this sense, these X-ray spectroscopies including XAS, HERFD-XAS, XES, RIXS and XPS through probing their specific energy-dependent inherent resonance can realize a comprehensive understanding of various dynamic events. Most notably, the RIXS technique can probe those electronic transition of a few eVs (at UV–vis region) by utilizing hard X-ray irradiation without requiring specific in situ cell, which reveals unprecedented and complementary information regarding the valence shell. Significantly, in addition to such a spectroscopy map, we have to stress that those techniques that are competent to track structural features of catalyst materials, such as X-ray scattering/diffraction/reflectivity and electron-based microscopies (liquid phase-TEM and scanning probe microscopy)^2,9^, are indispensable tools to complement the spectroscopy. From such a complete characterization map, an overall picture of target electrocatalyst with complex interactions at the solid–liquid interface can be intuitively understood to deal with the challenging dynamic configurations and to offer rational models for further theoretical calculations. We believe that such probing energy-dependent “in situ spectroscopy map” not only offers an indispensable in situ research model for future studies in the electrocatalysis field, but also provides unprecedented clues about novel catalysts design, which should have a far-reaching impact on numerous heterogeneous catalysis.

## Challenges of advanced X-ray spectroscopies

The advent of high brilliance synchrotron sources has triggered rapid development and employment of photon-hungry techniques like HERFD-XAS, XES and RIXS to probe structural and electronic configurations in a wide range of systems. However, with the increase in beam brightness, radiation/beam damage on samples cannot be ignored and deserves more attention for in situ/operando studies, because this issue leads to nonlinear effects to distort the electronic/geometric structures or even sample damaging that are also electrochemical reaction related. This is in particular the case for X-ray sensitive systems, such as metal–organic complexes (e.g., high-valent metalloproteins and metal complexes) in physiological or in operando conditions^99^. Recently, it has been demonstrated that even some transition-metal oxide systems are also sensitive to radiation damage during RIXS measurements with a relatively low soft X-ray dose^100^. Such situation has become a pressing challenge for performing X-ray spectroscopy studies on those biomimetic complexes and transition-metal oxides that are promising (electro)catalyst materials for improving energy applications. Thus, one must be aware of the possibility of radiation damage during X-ray spectroscopy measurements in order to avoid data that is contaminated by damage artifacts. Generally, radiation sensitivity is sample-, environment (e.g., electrolyte)- and instrumentation (photon energy, total deposited dose and beam size)-dependent^93^. During in situ/operando measurements, one can monitor the radiation effects in two ways: globally by measuring the electrochemical performance of the system or locally by X-ray imaging the single particle domain. If the radiation damage occurs, it can be mitigated by adapting environment or instrumentation conditions. Most notably, HERFD-XAS, XES and RIXS are photon-hungry techniques such that the radiation damage issue cannot be simply mitigated by reducing the photon flux. An efficient way to avoid radiation damage is quantifying the timescale required to obtain a difference between spectra by performing X-ray spectra centered on the edge energy^38^. This timescale is then the time limit before the appearance of radiation damage, and thus it is suggested that the spectrum’s duration during measurements should be shorter than the time limit. In this regard, another promising way is to improve detection efficiency, allowing X-ray spectroscopy experiments accomplished within a reasonable timescale. To this end, time-resolved experiments have been particularly proposed at synchrotron radiation light sources and for XFELs^75^. Since XFELs generate intense and femtosecond long pulses at very high repetition rates, X-ray spectroscopy at XFELs can be used to study the configuration dynamics of the radiation-sensitive system in the femtosecond time domain prior to the photodamage, yielding damage-free information, which represents a “probe-before-destroy” approach^101^. In addition, because of beam-induced photoreduction, collecting spectra data under cryogenic conditions is another strategy to significantly reduce the spurious effects induced by the X-ray. To fulfill such condition, spectra acquisition is frequently performed on frozen samples placed in an appropriate cryostat (liquid nitrogen or liquid helium). Besides, it should be noted that, if several spectra are necessary, such as several acquisitions for improving the data quality, the position of the beam should be changed on the sample between each scan to probe a fresh area.

For electrocatalytic systems, the reactions mainly occur within ~1 nm around the solid–liquid interface, suggesting that only atoms on the topmost layers of electrocatalyst directly mediate the catalytic processes, thus the surface signal is usually the most meaningful based on surface-sensitive techniques. Although soft X-ray XAS is more surface-sensitive as compared with hard X-ray XAS, it is still a bulk-sensitive technique because the penetration depth of soft X-ray is still in the micrometer range, and spectroscopies based on soft X-ray are ranging from a probing depth of hundreds of nanometers to even micrometers depending on the chosen edge. Recently, grazing incidence XAS is utilized to in situ reveal the structural evolution of the near-surface region of polycrystalline metal electrodes during electrocatalytic reactions^102,103^. The advantage of this approach, particularly for thin film, is the limited penetration depth of the X-rays into the sample, by varying the incident angle, the penetration depth can be changed from a few nanometers up to 100 nanometers. To realize the surface-sensitive probing, instead of measuring the emitted fluorescence, one can collect the emitted Auger electrons (the so-called total electron yield mode, TEY). Because of the short mean free path (~10 Å) of the Auger electrons emitted, the detected signal is significantly limited to the sample surface^104^. For instance, in situ XAS in TEY mode has been strongly demonstrated to investigate the local structure of interfacial water molecules near gold electrodes and its bias dependence^84^. Moreover, the fluorescence and total electron yield modes yield distinct spectra, elucidating the surface sensitivity of the TEY mode and strong interaction between the water molecules and the gold electrode at the interface. Nonetheless, the TEY measurements require UHV for the electron detection, and thus limiting its applicability for in situ/operando investigations of electrocatalytic processes. In particular, since the electron excited by X-ray could be interfered by applied voltages during the electrochemical reactions, advanced in situ cell designed to work in electron yield mode should be highly imperative.

## Summary and outlook

As discussed in this perspective, the electrochemical process over a real complex catalyst is considerably complicated at the solid–liquid interface under realistic conditions. Profound understandings of dynamic configurations toward a target electrocatalytic reaction are strongly dependent on both precise data interpretation and further advanced operando characterizations. Herein, one of the most important messages is that, for widely used XAS method, data analysis and interpretation for XANES and EXAFS spectra are often nontrivial and may result in misleading information. Especially, under operando electrochemical conditions, the disordered nature of the solid–liquid interface and dynamic transformations often make interfacial features intricate, which requires an appropriate fitting and scrupulous extraction of structural parameters. This perspective provides some practical guides for precisely determining oxidation state from XANES spectra and for conducting multi-shell analysis with an emphasis on the correlation problems of extracted parameters in EXAFS fitting process. On the other hand, an ongoing trend is the development of simulation-based approaches for XAS data interpretation^105^, such as machine learning and artificial neural networks^106,107^, which allows one to utilize artificial intelligence to rationally link the theoretical models with spectral features. For instance, by employing some machine learning methods, quantitative analysis of XANES spectra of metal–organic framework (MOF) CPO-27-Ni can be well performed^108^, which precisely predicts the geometry of adsorbed CO_2_ molecule on Ni^2+^ surface sites hosted in the channels of MOF materials. Such machine learning methods show disruptive potential to revolutionize the field of XAS researches^109^. Nonetheless, we have to point out that, by performing XAS only, it is unlikely to provide solid evidence for proposing reliable configurations, we do strongly suggest to conduct multimodal characterizations.

We particularly highlight several promising and innovative in situ/operando X-ray spectroscopies toward interfacial electrocatalysis, including HERFD-XAS, XES and RIXS. A few related studies have evidenced that they are emerging to be powerful techniques for giving new and unprecedented insights toward the dynamic interfacial configurations and reaction mechanism that are not accessible by conventional XAS approach. In addition to OER, ORR, HER and CO_2_RR, we can anticipate that these X-ray spectroscopies would be the most efficient and indispensable techniques to provide direct experimental proof for most (electro)catalytic reactions involving water, carbon and nitrogen cycles, such as hydrogen oxidation reaction (HOR)^110,111^, methanol/ethanol oxidation reaction (MOR/EOR)^112,113^, benzyl alcohol oxidation^114^, CO oxidation/reduction reaction (COOR/CORR)^62,115^, urea oxidation reaction (UOR)^116^, and nitrate reduction^117^ etc., although most results are currently based on the conventional XAS studies. More promisingly, these modern X-ray spectroscopies are expected to be powerful and employed in studies regarding energy storage/conversion devices, such as various fuel cells (e.g., hydrogen, alcohol and other small organic molecule fuel cells)^118,119^ and batteries (e.g., Li-ion, Mg-ion, Zn-ion, Ca-ion, Li-S, and Na-ion batteries)^93,94^. Because most intercalation-type cathodes in battery materials are made of 3*d* transition metals, in situ/operando hard X-ray HERFD-XANES, XES and RIXS approaches are ideal platforms to reveal the electronic evolutions and structural changes of those 3*d* transition metals and corresponding charging/discharging dynamics in battery electrodes. Accordingly, we can envision the rising significance and necessity of these promising X-ray spectroscopies in the (electro)catalysis field.

Moreover, we encourage that the time-resolved X-ray spectroscopies, in which the timescale of acquiring spectrum matches well with realistic electrochemical reactions, should be fully developed and utilized to realize real-time tracking of dynamic changes in atomic configurations during reactions. Recently, the advent and development of X-ray free-electron lasers announce highly coherent, intense and short-pulsed X-ray beams, which advances the exploration of matter dynamics at atomic scales with femtosecond time resolution and inspires the ultrafast time-resolved experiments in electrocatalysis^120^. Additionally, another issue has to be emphasized the importance of monitoring dynamic changes of catalysts in long duration that is crucial to validate the practically catalytic fate for industrial applications^121^. Considering that long-term characterizations under working conditions (up to several weeks) are not practical for academic institutions, the next step may be to develop accelerated tests or degradation protocols for addressing the benchmark catalyst stability.

Finally, to achieve a comprehensive picture of the working interface during reactions, we propose the complementarity from hard and soft X-ray spectroscopies with coupling various characterization techniques. In particular, a probing energy-dependent “in situ spectroscopy map” is well established and suggested to provide highly complementary information about the dynamic configuration of electrochemical interfaces. An integration of advanced X-ray spectroscopies with various complementary techniques will be without a doubt the wave of the future. Fully complementary information obtained from various perspectives provided by several approaches will be the only way to unambiguously answer the questions about those intricate (electro)catalytic processes that are taking place at the solid–liquid interface. Overall, the present perspective will inspire numerous follow-up studies to employ advanced in situ/operando techniques to make great contributions in both electrocatalysis and characterization techniques.
